# Supplementary figures and images for: Non-fatal injuries in rural Burkina Faso amongst older adults, disease burden and health system responsiveness: a cross-sectional household survey
Source: BMJ Open. 2021 May 28;11(5):e045621. doi: 10.1136/bmjopen-2020-045621 (PMC8166610; doi:10.1136/bmjopen-2020-045621)

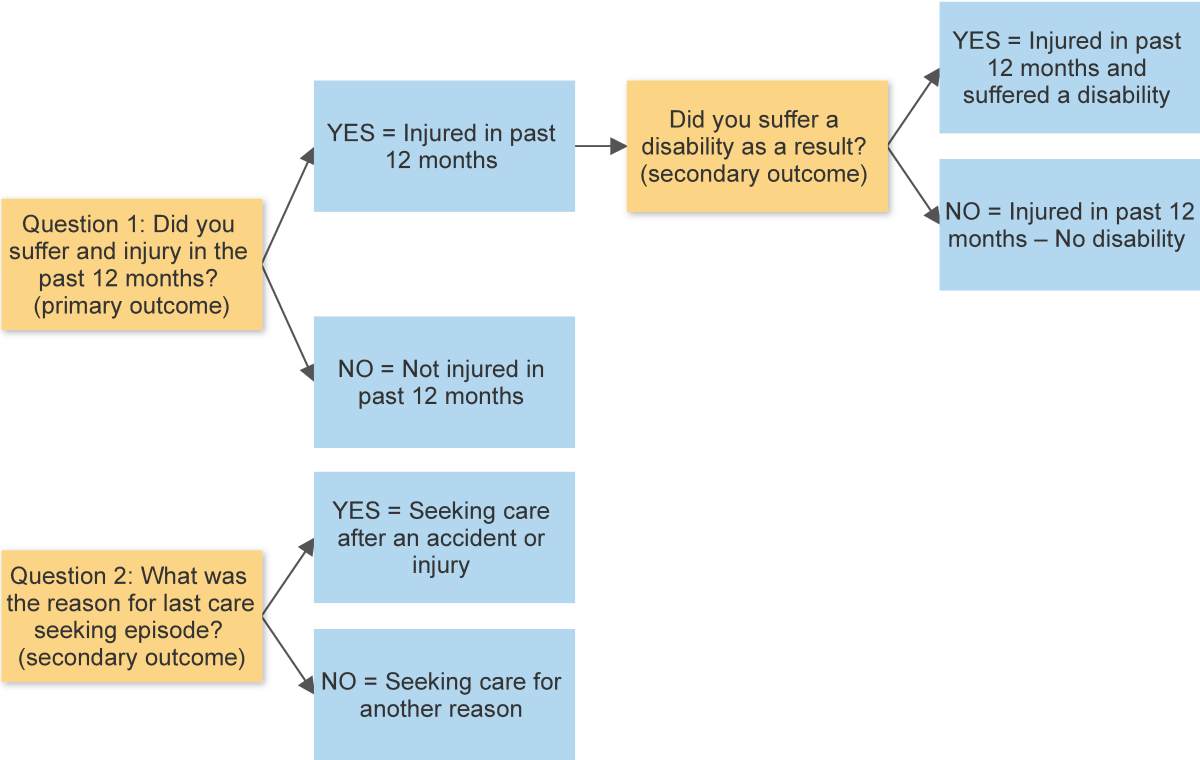

Supplement: Supplementary data [file bmjopen-2020-045621supp001.pdf]
