# Supplementary material for: Non-fatal injuries in rural Burkina Faso amongst older adults, disease burden and health system responsiveness: a cross-sectional household survey
Source: BMJ Open. 2021 May 28;11(5):e045621. doi: 10.1136/bmjopen-2020-045621 (PMC8166610; doi:10.1136/bmjopen-2020-045621)
Supplement: Supplementary data [file bmjopen-2020-045621supp002.pdf]

Appendices

Appendix table 1 - Mechanism of injury according to wealth quintile, for all those injured in past 12 months with number of males in brackets.

Wealth Quintile	Fall N (n of males)	Fall from motorcycle or bicycle N (n of males)	Struck / hit by object N (n of males)	Cut / stabbed N (n of males)	Gunshot N (n of males)	Fire / Heat Burn N (n of males)	Poisoning N (n of males)	Animal bite N (n of males)	Other N (n of males)
1	6 (4)	3 (3)	10 (7)	11 (5)	1 (1)	1 (0)	0 (0)	2 (1)	4 (2)
2	5 (1)	6 (5)	17 (13)	2 (1)	2 (2)	1 (1)	3 (0)	1 (1)	0 (0)
3	9 (5)	12 (6)	12 (8)	15 (11)	0 (0)	0 (0)	1 (1)	0 (0)	3 (2)
4	3 (3)	13 (10)	20 (11)	5 (5)	0 (0)	2 (0)	0 (0)	2 (2)	3 (1)
5	3 (2)	19 (15)	17 (9)	13 (8)	1 (0)	1 (0)	0 (0)	3 (2)	0 (0)
Total	26 (15)	53 (39)	76 (48)	46 (30)	4 (3)	5 (1)	4 (1)	8 (6)	10 (5)

Appendix table 2 - Binary logistic regression for falling from a motorcycle or bicycle and wealth quintile.

	Odds Ratio	95% C.I.	P Value
Quintile 1 (ref)			
Quintile 2	2.26	0.52 - 9.80	0.277
Quintile 3	3.50	0.91 - 13.42	0.068
Quintile 4	4.33	1.14 - 16.55	0.032
Quintile 5	5.83	1.59 - 21.43	0.008

Appendix table 3 - Opinion and experience of healthcare received by those seeking care following an injury and those seeking care for another reason at last visit.

	Seeking care after an accident or injury % (95% CI)<br>Number responders (N) = 119 unless otherwise stated	Seeking care for another reason n % (95% CI)<br>Number responders (N) = 2882 unless otherwise stated
**Opinion of care received**		
Confident that would receive effective treatment if very sick tomorrow		
Very or somewhat confident	95.8 (90.5 – 98.2)	93.5 (92.6 – 94.4)
Not very or not at all confident	4.2 (1.8 – 9.5)	6.5 (5.6 – 7.4)
Overall view of national healthcare system		
Needs to be rebuilt or major changes needed	46.2 (37.5 – 55.2)	38.0 (36.3 – 39.8) (N=2909)
Only minor changes needed	53.8 (44.9 – 62.5)	62.0 (60.2 – 63.7) (N=2909)
Overall how well did received care meet health needs at last visit?		
Excellent, very good or good	90.0 (83.2 – 94.1)	94.4 (93.5 – 95.2)
Fair or poor	10.0 (5.9 – 16.8)	5.5 (4.8 – 6.5)
Do you trust in the skills and abilities of the HCW at the facility?		
Very much, quite a bit or some.	96.6 (91.7 – 98.7)	97.7 (97.1 – 98.2) (N=2879)
Very little or not at all	3.4 (1.3 – 8.3)	2.3 (1.8 – 2.9) (N=2879)
What is your opinion of the care provider's knowledge and skills?		
Excellent, very good or good	95.0 (89.4 – 97.7)	94.5 (93.3 – 95.0) (N=2873)
Fair or poor	5.0 (2.3 – 10.6)	5.5 (5.0 – 6.7) (N=2873)
What do you think about the provider's ability to explain things in a way that you could understand?		

Whitaker J, et al. BMJ Open 2021; 11:e045621. doi: 10.1136/bmjopen-2020-045621

	Excellent, very good or good	95.0 (89.4 – 97.7)	94.2 (93.3 – 95.0) (N=2864)
	Fair or poor	5.0 (2.3 – 10.6)	5.8 (5.0 – 6.7) (N=2864)
How easy or difficult was it for you to follow the provider's advice?			
	Very Easy, easy or fair	99.2 (95.4 – 99.9)	97.8 (97.3 – 98.3) (N=2877)
	Hard or very hard	0.8 (0.2 – 4.6)	2.2 (1.7 – 2.8) (N=2877)
What do you think about your experience of being involved in making decisions for your treatment?			
	Excellent, very good or good	74.3 (65.6 – 81.5) (N=113)	77.7 (76.1 – 79.2) (N=2748)
	Fair or poor	25.7 (18.5 – 34.4) (N=113)	22.3 (20.8 – 23.9) (N=2748)
**Experience of care processes**			
Did care provider refer to another facility? - Yes		9.3 (5.2 – 15.8)	5.4 (4.6 – 6.3) (N=2872)
Did care provider ask for further tests? - Yes		10.3 (6.0 – 17.1) (N=117)	7.8 (6.9 – 8.8) (N=2859)
Did the care provider ask you to come back? - Yes		81.4 (72.7 – 86.8)	51.8 (50.0 – 53.6) (N=2881)
Had to borrow or sell to pay for health care episode? - Yes		21.2 (14.7 – 29.7) (N=113)	14.3 (13.0 – 15.6) (N=2881)
How long did you wait before your consultation (minutes)? (Median and IQR) (Mann-Witney U)		10 (5-15) (N=118)	20 (10-30) (N=2885)<br><br>P = 0.002
How much time did you spend with the care provider (minutes)? (Median and IQR) (Mann-Witney U)		20 (15-30) (N=118)	15 (20-25) (N=2885)<br><br>P = 0.002

Whitaker J, *et al. BMJ Open* 2021; 11:e045621. doi: 10.1136/bmjopen-2020-045621
