# Supplementary material for: Non-fatal injuries in rural Burkina Faso amongst older adults, disease burden and health system responsiveness: a cross-sectional household survey
Source: BMJ Open. 2021 May 28;11(5):e045621. doi: 10.1136/bmjopen-2020-045621 (PMC8166610; doi:10.1136/bmjopen-2020-045621)
Supplement: Author's manuscript [file bmjopen-2020-045621.draft_revisions.pdf]

BMJ Open is committed to open peer review. As part of this commitment we make the peer review history of every article we publish publicly available.

When an article is published we post the peer reviewers' comments and the authors' responses online. We also post the versions of the paper that were used during peer review. These are the versions that the peer review comments apply to.

The versions of the paper that follow are the versions that were submitted during the peer review process. They are not the versions of record or the final published versions. They should not be cited or distributed as the published version of this manuscript.

BMJ Open is an open access journal and the full, final, typeset and author-corrected version of record of the manuscript is available on our site with no access controls, subscription charges or pay-per-view fees (http://bmjopen.bmj.com).

If you have any questions on BMJ Open's open peer review process please email info.bmjopen@bmj.com

BMJ Open

Non-fatal injuries in rural Burkina Faso, disease burden and health system responsiveness, a household survey.

Journal:	*BMJ Open*
Manuscript ID	bmjopen-2020-045621
Article Type:	Original research
Date Submitted by the Author:	12-Oct-2020
Complete List of Authors:	Whitaker, John; King's College London Faculty of Life Sciences and Medicine, Centre for Global Health and Health Partnerships; Royal Centre for Defence Medicine, Academic Department of Military Surgery and Trauma<br>Harling, Guy; University College London, Institute for Global Health; Harvard University T H Chan School of Public Health, Department of Epidemiology<br>Sie, Ali; Centre de Recherche en Sante de Nouna<br>Bountogo, Mamadou; Centre de Recherche en Sante de Nouna, Nouna; Université de Ouagadougou, Ouaga<br>Hirschhorn, Lisa; Northwestern University Feinberg School of Medicine, medical Social Sciences; Northwestern University Feinberg School of Medicine,<br>Manne, Jen; Harvard Medical School, Division of infectious diseases<br>Bärnighausen, Till ; Ruprecht Karls University Heidelberg Faculty of Medicine Heidelberg, Institute of Global Health<br>Davies, Justine ; University of Birmingham Institute of Applied Health Research; University of the Witwatersrand School of Public Health, MRC/Wits Rural Public Health & Health Transitions Research Unit (Agincourt
Keywords:	Trauma management < ORTHOPAEDIC & TRAUMA SURGERY, HEALTH SERVICES ADMINISTRATION & MANAGEMENT, EPIDEMIOLOGY, TROPICAL MEDICINE

SCHOLARONE™
Manuscripts

I, the Submitting Author has the right to grant and does grant on behalf of all authors of the Work (as defined in the below author licence), an exclusive licence and/or a non-exclusive licence for contributions from authors who are: i) UK Crown employees; ii) where BMJ has agreed a CC-BY licence shall apply, and/or iii) in accordance with the terms applicable for US Federal Government officers or employees acting as part of their official duties; on a worldwide, perpetual, irrevocable, royalty-free basis to BMJ Publishing Group Ltd ("BMJ") its licensees and where the relevant Journal is co-owned by BMJ to the co-owners of the Journal, to publish the Work in this journal and any other BMJ products and to exploit all rights, as set out in our licence.

The Submitting Author accepts and understands that any supply made under these terms is made by BMJ to the Submitting Author unless you are acting as an employee on behalf of your employer or a postgraduate student of an affiliated institution which is paying any applicable article publishing charge ("APC") for Open Access articles. Where the Submitting Author wishes to make the Work available on an Open Access basis (and intends to pay the relevant APC), the terms of reuse of such Open Access shall be governed by a Creative Commons licence – details of these licences and which Creative Commons licence will apply to this Work are set out in our licence referred to above.

Other than as permitted in any relevant BMJ Author's Self Archiving Policies, I confirm this Work has not been accepted for publication elsewhere, is not being considered for publication elsewhere and does not duplicate material already published. I confirm all authors consent to publication of this Work and authorise the granting of this licence.

For peer review only

Title: Non-fatal injuries in rural Burkina Faso, disease burden and health system responsiveness, a household survey.

Authors

John Whitaker 1,2 – corresponding author
Guy Harling 3,4,5,6
Ali Sie 7
Mamadou Bountogo 7
Lisa R Hirschhorn 8
Jennifer Manne-Goehler 9
Till Baernighausen 3, 10
Justine Davies 5, 11

Affiliations

1 - King's Centre for Global Health and Health Partnerships, School of Population Health & Environmental Sciences, Faculty of Life Sciences and Medicine, King's College London, London, UK. Room 2.13, Global Health Offices, Weston Education Centre, Cutcombe Road, London, SE5 9RJ, UK. john.k.whitaker@kcl.ac.uk.
2 - Academic Department of Military Surgery and Trauma, Royal Centre for Defence Medicine, Birmingham, UK
3 - Africa Health Research Institute, KwaZulu-Natal, South Africa.
4 - Institute for Global Health, University College London, London.
5 - MRC/Wits Rural Public Health & Health Transitions Research Unit (Agincourt), University of the Witwatersrand, Johannesburg-Braamfontein, South Africa
6 - Department of Epidemiology & Harvard Center for Population and Development Studies, Harvard T.H. Chan School of Public Health, Boston, MA, USA
7 - Centre de Recherche en Sante de Nouna, Nouna, Burkina Faso
8 - Department of Medical Social Sciences, Feinberg School of Medicine, Northwestern University, Chicago, IL, USA.
9 - Division of Infectious Diseases, Massachusetts General Hospital, Harvard Medical School, Boston, Massachusetts, USA
10 - Heidelberg Institute of Global Health (HIGH), Faculty of Medicine and University Hospitals, University of Heidelberg, Heidelberg, Germany
11 - Centre for Applied Health Research, University of Birmingham, Birmingham, UK.

For peer review only

Abstract:

Introduction
Injury is an important cause of avoidable death and disability with sparse research in low-income countries. This study aimed to evaluate injury epidemiology and patient-reported health system responsiveness in older individuals in rural Burkina Faso.

Methods
A cross-sectional household survey of adults over 40 from the Nouna Health and Demographic Surveillance System collected data on experience of injury in past 12 months. This included mechanism and associated disability, reasons for last health facility visit, health system responsiveness, anxiety and depression, quality of life and frailty. Multivariable analyses determined associated factors and consequences of injury and related disability. Univariable comparison determined differences in health system responsiveness for those seeking care following injury and another reason.

Results
7.7% (232/3028) reported injury in the preceding 12 months. In multivariable analyses, younger age, male sex, highest wealth quintile, an abnormal Generalised Anxiety Disorder score and lower WHO QOL score were all associated with injury. The most common mechanism of injury was being struck or hit by an object, 32.8%. In multivariable analysis only education was significantly negatively associated with odds of disability (OR 0.407, 95%CI 0.17–0.997). Across all survey participants 3.9% reported seeking care last following injury. Positive experience and satisfaction with care were reported following injury. Injury care had shorter median wait times (10 vs 20 minutes, P=0.002) and longer consultation times (20 vs 15 minutes P=0.002) than care for another reason. Injured patients were also asked to return to health facilities more often, 81.4% (95%CI 73.1%–87.9%) vs. 54.8% (95%CI 49.9%–53.6%).

Conclusion
Injury is an important disease burden in this older adult rural LMIC population. Further research could inform preventative strategies including safer rural farming methods, explore the association between adverse mental health and injury, and strengthen health system readiness to provide quality care.

Key words
Wounds and injuries, developing countries, health services research, epidemiology, surveys and questionnaires.

Word count
4073

Article Summary

Strengths and limitations of this study

- We were able to establish the size of non-fatal injury burden in rural Burkina Faso, where little empirical data on injury exists.
- We were able to explore associations with psychological morbidity and quality of life for those reporting injuries, and understudied aspect of LIC injury burden.
- Reporting he most recent reason for accessing care allowed us to compare health system responsiveness following injury with other conditions.
- The survey lacked clear definitions of injury and disability which may have led to overestimation of burden.

For peer review only

Author Contributions

TB, AS and GH conceived and designed the overall CSRN CHAS study.

MB and GH co-ordinated baseline data collection and preparation.

JID, JM-G and LRH contributed to the design of the CSRN CHAS household survey.

JW and JID designed the current study.

JW conducted the analysis, wrote and revised the manuscript.

JID supervised the analysis, write up and development of the manuscript.

All authors substantively reviewed manuscripts, inputted into revisions and approved the final manuscript.

Funding statement

Funding Support for the CRSN Heidelberg Aging Study and for TB was provided by the Alexander von Humboldt Foundation through the Alexander von Humboldt Professor award (no grant number exists) to Till Bärnighausen, funded by the German Federal Ministry of Education and Research. GH is supported by a fellowship (210479/Z/18/Z) from both the Wellcome Trust and Royal Society. JMG was supported by Grant Number T32 AI007433 from the National Institute of Allergy and Infectious Diseases. JW is a serving member of the UK Defence Medical Services.

Competing interests: - None declared.

Patient and public involvement: - Patients and/or the public were not involved in the design, or conduct, or reporting or dissemination plans of this research.

Patient consent for publication: - Not required.

Ethics approval: - Ethical approval was obtained from Ethics Commission I of the medical faculty Heidelberg (S-120/2018), the Burkina Faso Comité d'Ethique pour la Recherche en Santé (CERS) in Ouagadougou (2018-4-045) and the Institutional Ethics Committee (CIE) of the CRSN (2018-04). Oral assent was sought from all village elders. Written informed consent was obtained from each participant and a literate witness assisted in cases of illiteracy

Data availability statement: - Data may be obtained from a third party and are not publicly available. Data are not publicly available as consent was not given by participants for this to take place. This is in part because entire age cohorts of some villages are included in the data set, potentially allowing for deductive disclosure with sufficient local information. For this reason, anonymised data are available from CHAS study data controllers only following signature of a data use agreement restricting onward transmission. Anyone wishing to replicate the analyses presented or conduct further collaborative analyses using CHAS (which are welcomed and considered based on a letter of intent), should contact Dr Guy Harling (g.harling@ucl.ac.uk) in the first instance.

For peer review only

Introduction

Injury is a neglected but important cause of avoidable disability and causes more than five million deaths globally every year [1], more than tuberculosis, malaria and HIV combined. Low and Middle Income Countries (LMICs) disproportionately bear this burden with 90% of global injury related deaths [2]. However injury related deaths are only the tip of the iceberg with an estimated one billion people sustaining injuries that require healthcare annually [1].

Burkina Faso is a landlocked country of 19 million people in sub-Saharan Africa. It is a low-income country (LIC) ranked 183 of 189 countries on the Human Development Index [3] with limited natural resources [3]. 2017 GBD estimates across all age groups that injuries are responsible for 7.32% of deaths and 6.48% of DALYs in Burkina Faso, similar to sub-Saharan Africa rates [4].

In common with the least developed countries, research investigating injuries in Burkina Faso has been sparse [5]. Studies that have been conducted tend to be referral facility based often with small case numbers and limited to a single mechanism of injury pattern [6-10]. Road traffic collisions (RTCs) have been investigated specifically and the World Health Organisation (WHO) has estimated that in 2016 there were 30.5 per 100,000 road traffic fatalities in Burkina Faso, above the average for Africa (26.6) which is the continent with the highest death rate globally [11]. RTC victim data from the tertiary referral hospital in the capital Ouagadougou identified that 87% of road traffic victims attending for tertiary care emergency department were from two wheel motor vehicles; a quarter of these experienced disability beyond 30 days[12].

Broader injury epidemiology studies from Burkina Faso have primarily studied cause of death data obtained through Verbal Autopsy. In the capital, Ouagadougou, a survey within an urban Health and Demographic Surveillance System (HDSS) identified 4.1% of deaths were due to injury [13]. From the rural Nouna HDSSs Verbal Autopsy data, age and sex standardised mortality for external causes of death (the category containing injuries) was almost twice that of the urban comparator in Ougadougou, with the main cause being transport related. Unfortunately, these Verbal Autopsy based surveys do not capture all mechanisms of injury, and they do not allow assessment of non-fatal injury occurrence [14]. Injury has also been characterised as a disease affecting the young, and some population studies of injury in sub-Saharan Africa have even excluded adults over 70[15]. However older people represent an important and growing population in LMICs. How and why older people are injured and the consequences associated with these injuries require further exploration.

Injuries can have a lasting impact on the victims through physical disability, previously shown beyond 30 days in over a quarter of RTC victims in Ouagadougou,[12] but also psychological morbidity. From High-income country (HIC) settings depression, anxiety and post-traumatic stress are commonly associated with physical injury[16 17]. This includes older populations with worse quality of life, psychological and social health status seen following hip fractures and osteoporotic vertebral fractures[18 19]. Poor mental health is also a risk factor for non-accidental injuries [20 21]. The impact of mental health following injury within Burkina Faso amongst general older adult population has not been studied, with mental health studies limited to vulnerable populations such as sex workers and children exposed to physical violence in Burkina Faso[22 23].

It has been estimated that if LMIC injury care quality could match that of HIC then 1/3 of all trauma deaths could be avoided [24], It is thus necessary to improve injury epidemiology data from Burkina Faso to inform preventative measures and treatment services. However, provision of care alone may not be associated with improved outcomes. Such care needs to be responsive to patients needs beyond providing good clinical outcomes in order to engender trust leading to compliance with treatment and encouragement of future injured persons to attend services [25-27]. However very few studies on responsiveness of injury care have been done in LMICs [28].

This study aimed to evaluate the epidemiology of injury as well as patient-reported health system responsiveness following injury and how this compares with non-injured patient experience, in a population of older individuals in rural Burkina Faso.

For peer review only - http://bmjopen.bmj.com/site/about/guidelines.xhtml

Methods

We used the STROBE cross-sectional reporting guidelines [29].

Study setting

The study was set in the Nouna HDSS area, in the Boucle du Mouhoun region, north-western Burkina Faso. The HDSS collects annual birth, death and migration data in a well-enumerated population. The HDSS area consists of the market town Nouna and 59 surrounding villages with a total population of around 107,000 [30]. Residents come from multiple ethnic groups and the major economic activities are farming and animal husbandry. Life expectancy from birth is 58.0 years for men and 61.5 years for women [31]. There is one tarred road running through the area. There are no formal ambulances with emergency transport usually informal via private or taxi motorbike. In rainy season travel can be very difficult.

Study design

This study is an analysis of the CRSN Heidelberg Aging Study dataset (CHAS). The study methodology has been described in detail elsewhere [32 33] . Briefly, this cross-sectional study consists of a population-representative sample of adults ≥40 years of age. 3000 older adults were randomly sampled from the 2015 Nouna HDSS census. In all villages (n=6) with fewer than 50 adults aged over 40, all adults were selected to take part. In all other villages, a random sample of households with at least one person over 40 years old was drawn, and then within each selected household one age-eligible adult was randomly selected to complete the survey. Data collection was performed using Open Data Kit (ODK) software on tablet computers at the participants' residence between May and July 2018[34]. Interviews were conducted either in French or translated into Dioula by the interviewers.

Variables

The household survey contained questions on age, sex, education, marital status, household assets, experience of injury in past 12 months including mechanism and associated disability, reasons for last health facility visit, and questions covering the WHO health system responsiveness domains [25] derived from other surveys used in sub-Saharan Africa [35-37]. Anxiety was assessed using the Generalised Anxiety Disorder question (GAD-2) score, [38] and depression using Patient Health Questionnaire (PHQ- 9) [39 40]. Quality of life was measured using the validated EuroHIS 8-item version of WHOQOL[41 42]. Disability was measured using the 12 item WHO Disability Assessment Schedule, version 2 (WHODAS-II) disability score [42 43]. The Fried frailty score was constructed from questions on weight loss in the past year, self-reported activity and levels of exhaustion, combined with measures of walking speed and grip strength [44 45].

Outcome variables

The main outcome variables were whether injured or not in the preceding 12 months, or if injured, whether disabled as a result of the injury. Participants reported whether they had any event where they suffered from bodily injury in the last 12 months. For those reporting yes, the cause of injury was reported along with the question "did you suffer a physical disability as a result of being injured?".

Mechanism of injury

Mechanism of injury was captured as either fall, struck/hit by object, cut/stabbed, gunshot, fire/heat burn, drowning/near-drowning, poisoning, animal bite, electric shock or other specified by free text. Injury mechanisms with fewer than 8 or cases were combined as "other" for analyses. Those who fell reported whether this was at or higher than ground level.

Demographic characteristics

Marital status was categorized as married/cohabiting or single/widowed/divorced. Educational level was categorized as no education or any education. Participants were asked 37 questions on household assets and dwelling characteristics; from these, wealth quintiles were derived from the Filmer and Pritchett first principal component method[46].

Definitions of disease states

Participants were defined as having symptoms of anxiety based on a GAD-2 score ≥3 [38]. Participants scoring 10 or more on PHQ-9 were categorized as having depressive symptoms in this analysis[40]. The calculation of the Fried score used in this study has been described previously [32]. For this analysis participants were dichotomised as robust or prefrail/frail. WHODAS-II disability score was

For peer review only - http://bmjopen.bmj.com/site/about/guidelines.xhtml

normalised to a 0-100 scale, where 0 equates to no disability and 100 the worst disability. Quality of life[41 42] was similarly normalised to a 0-100 scale, with 100 denoting best quality of life.

Health system experience and responsiveness

Regardless of when it occurred, the reason for the most recent episode of health seeking was recorded and classified as either accident/injury or another reason. Participants answering this question were not necessarily the same as those injured in the previous 12 months who may have sought care for another reason subsequent to their injury. Those who had sought care were asked health system responsiveness questions, including: (i) confidence in receiving effective treatment if very sick tomorrow, dichotomised as very/somewhat vs. not very/not at all; (ii) the overall view of the health system, dichotomised as needs to be rebuilt/major changes needed vs. only minor changes needed; (iii) trust in the skills and abilities of the healthcare worker at the facility dichotomised as 1. very much, quite a bit or some and 2. very little or not at all; (iv) ease or difficulty in following provider's advice dichotomised as 1. very easy, easy or fair and 2. hard or very hard; and (v) opinion of care provider's knowledge and skills, experience of being involved in making decisions for treatment, ability of provider to explain things in a way they could understand and how well the received care met health needs were all dichotomised into positive responses 1. excellent, very good, or good and negative responses 2. fair or poor. These variables were dichotomised for ease of interpretation.

Patient and public involvement statement

Participants were not directly involved in planning the study; results of this and other HDSS studies are regularly fed back to participants in the HDSS site.

Statistical analysis

All analyses were done using SPSS v26 (IBM, New York, USA). We first described all variables using mean and standard deviation (SD), or median and interquartile range (IQR), for normally and non-normally distributed continuous variables, and count and proportion (95% CI) for categorical variables.

We used multivariable logistic regression to explore the associations between the main outcome variables and demographic characteristics or disease states. Odds ratios and 95% confidence intervals are presented. All variables were included in the model. Figures were produced using the R package ggplot2[47].

Associations between seeking care for an accident/injury or another reason and healthcare experience and health system responsiveness were tested using Mann-Whitney U test for the non-normally distributed continuous variables. Sample sizes are stated for each analysis.

For peer review only

Results:

The median age of respondents was 52 years (IQR 45 – 62), females made up 50.7% (1534/3028) of the population, educational attainment was low, with only 15.6% (472/3028) having any schooling at all (Table 1).

Of those completing the survey, 7.7% (232/3028) reported suffering an injury in the preceding 12 months (Table 1). In multivariable analyses, younger age, male sex, highest wealth quintile, an abnormal GAD score and lower WHO QOL score were all associated with injury (Table 2).

The most common mechanism of injury was being struck or hit by an object, 32.8% (76/232) (Figure 1). Of those who suffered a fall, 34.6% (9/26) fell from higher than ground level. Exploratory analysis of the association between mechanism of injury and wealth (appendix tables 1 and 2) suggested that the greater odds of being injured in the higher wealth quintile is related to a greater number of falls from a motorcycle or bicycle in this group; 35.8% (19/53) of those falling from a motorcycle or bicycle were from wealth quintile 5 compared to 5.7% (3/53) in quintile 1 (Odds ratio 5.83, 95% CI 1.58 - 21.43).

Falling from a motorcycle or bicycle was the mechanism which most frequently resulted in a disability 27.6% (29/105) (Figure 1). In multivariable analysis (Table 3) only education was significantly negatively associated with odds of disability (Odds ratio 0.407, 95% CI 0.17 – 1.00). Compared to being struck or hit by an object, disability was more common amongst those falling (odds ratio 6.4, 95% CI 1.896 – 21.602, p=0.003), falling from a motorbike (odds ratio 3.335, 95% CI 1.429 – 7.78, p=0.005), and other (odds ratio 10.755, 95% CI 3.471 – 33.323, p<0.001).

Across all survey participants 3.9% (119/3028) of people reported their last reason for seeking care was for an injury or accident. These 119 respondents reported high levels of satisfaction with care following injury (Figure 2). Ninety-six percent reported being somewhat or very confident of receiving effective treatment if sick tomorrow, 95% reported a good or better opinion of care provider's skills and knowledge and 90% reported that their needs were met well or better. There were no significant differences in these measures between people seeking care for injuries and those seeking care for other reasons (Figure 2 and appendix Table 3).

Those seeking care following injury reported shorter median wait times (10 minutes vs 20 minutes, P=0.002) before consultation and longer consultation times (20 minutes vs 15 minutes P=0.002) than those seeking care for another reason. Those seeking care for injury were also more likely to be asked to return to the health facility at a later date, 81.4% (95% CI 73.1% – 87.9%) vs. 54.8% (95% CI 49.9% – 53.6%) (Figure 2). There was a non-significant trend for those seeking injury care to be more likely to borrow or sell to pay for the care episode, compared to those seeking care for other reasons, 21.2% (95% CI 14.7% - 29.7%) vs. 14.3% (95% CI 13.0% - 15.6%).

For peer review only - http://bmjopen.bmj.com/site/about/guidelines.xhtml

Discussion:

This study demonstrates that injuries are prevalent in this older adult rural LMIC population. In this cross sectional study we cannot show causation, however, injuries were more prevalent in those who were male, or of younger age, or wealthier socio-economic status. Those with injuries were more likely to suffer from anxiety or depression, or report a worse quality of life. Almost half of those reporting an injury reported disability as a consequence, which was more common in males and those with lower educational attainment, but not associated with frailty. Patient satisfaction with the health system for treatment of an injury was generally high. Having to sell or borrow to pay for care was more common than for non-traumatic health care visits although this did not reach significance. There is little empirical data published on injury prevalence and care within Burkina Faso, particularly in older people. This study can aid researchers and policy makers in understanding the burden to address prevention and avenues for further research.

Although in an older population, this study found the incidence of injury, 7.7%, was comparable to other sub-Saharan African settings such as in rural Tanzania, rural Rwanda, rural Nigeria, Sudan, Sierra Leone and Kenya where studies have shown prevalence ranges from 4.3 – 15.2% [15 48-52]. Other studies from Sub-Saharan Africa have also found injuries to be more common in younger [15 52] or male [15 48 50 51] members of the population. Indeed, male sex is consistently associated with injury globally, with multiple possible contributing factors including alcohol use, dangerous occupations, or risk taking behaviour [53] – unfortunately none of these were evaluated in CHAS.

Globally, poorer populations bear increased injury burden [54] including amongst urban populations [51] and those sustaining accidental injury [15] perhaps due to those of lower SES being exposed to less safe working conditions. We found SES to be positively associated with injury occurrence; potentially, in this rural context, it is likely that relative wealth provided access to motorcycles or bicycles that may have been unaffordable for poorer groups.

Anxiety and reduced quality of life were associated with occurrence of injury although no association was seen with depression. Others have shown adverse mental health outcomes to be sequalae of physical injury and include post-traumatic stress disorder, depression and anxiety [16 17 55]. Whilst research exists in high income settings, further research into the adverse mental health associations with injury in this and other LMIC contexts is warranted to establish burden and direction of causality. Development of culturally specific tools for evaluating post physical trauma mental health in African populations is also justified [56].

Almost half those injured reported disability (although not defined) as a consequence of their injury, and reporting disability after injury was associated with lower educational status. Other sub-Saharan African studies have reported varying levels of disability after injury, for example, 31.7% in Rwanda [49], and 11% in Sudan [57]. Disability can be more prevalent in rural compared to urban settings [58], amongst the uneducated [57], and in adults over 60 [58]. The different questions employed in these studies makes direct comparison difficult although the high incidence of disability we found may be due to studying an older population with less physical reserve.

In our study, no association between disability and frailty was seen in the population who had been injured. A lower baseline physical function may affect the threshold for self-reported disability. The non-frail population, with low educational attainment, may also have been more dependent on physical labour than other studies and thus more sensitive to limitations to physical function. The association between disability and lower levels of education seen in our study supports this.

The most common injury mechanisms were being struck or hit by an object, falling from a motorcycle or bicycle, being cut or stabbed, or falling. In rural environments injuries can commonly be a consequence of agricultural activity. This was the leading contributor to injuries in rural Ghana [58]. In Tanzania cuts or stabs were the most common mechanisms in the rural population studied and two-thirds of cuts were due to agricultural activity [48]. Transport or road traffic accidents were not a distinct category within our study given that cars are rarely used in Nouna.

Interestingly, despite the older age of the CHAS population relative to most previous studies, falls were relatively uncommon, unlike has been reported in Kenya [15], Nigeria [50], and Sierra Leone [52] and especially in older persons in Tanzania (aged over 60) [48] Ghana (aged over 60)[58] and Sudan (aged over 45) [51]. The prevalence of frailty in this population is similar to that seen in other sub-

For peer review only - http://bmjopen.bmj.com/site/about/guidelines.xhtml

Saharan African populations where this has been studied, so the relatively low prevalence of falls (which are associated with frailty) need further investigation [32].

Patients reported a positive experience and high satisfaction with care for both injury and non-injury related consultations. Injured patients experienced shorter wait times and longer consultation time with more frequent requests to return to care than those presenting to healthcare facilities for other reasons. This possibly reflects the urgency of injury care and the need for procedural management such as suturing needing follow-up. Patient satisfaction is influenced by factors such as accessibility, cost, expectation, immediate outcomes and gratitude [28]. However, in LMICs, patient reported satisfaction may not correspond well with other measures of care input, process, or clinical outcome and has even been associated with poor technical quality care [28].

We didn't study care quality dimensions such as facility human and physical resource availability or trauma specific diagnostic and management processes. Other studies from sub-Saharan Africa have shown limitations in basic care delivery for injuries including analgesia and fracture splinting [59]. Trauma mortality in LICs is worse than middle and high income countries [24] likely reflecting available care quality. Many facilities lack minimum essential resources [60-63] and post-graduate injury care training is uncommon [64]. Care quality for other conditions has been assessed in Nouna. Video vignettes have been used to measure health care worker knowledge [65] and primary maternal and child health care quality has been assessed using facility surveys, direct observation of care, exit interviews, patient record review and maternal and child health registers [66] demonstrating care quality gaps even after intervention. Similar methods could be used in future to assess injury care quality further in this population.

As a time critical condition in an economically poor population there is a risk of injury causing impoverishment. Out-of-pocket expenditure for healthcare is commonplace in Burkina Faso [67]. The economic burden of trauma and injuries in LMICs is high through direct medical costs such as medicines, non-medical costs such as transport and indirect costs such as loss of income [68]. Significant economic benefit could derive from reducing injury burden [69]. In Burkina Faso, CT scan access in Ouagadougou was limited by lack of funding when indicated in 20% of cases[10]. Indeed, perceived and actual costs of care are a well noted factor in delaying access to quality care after injury [26 27 49 57 70]. In CHAS, we found a non-significant trend that patients more commonly needed to borrow or sell following injury than other conditions. This could be compounded by the effect of injury related disability on economic productivity. While CHAS did not capture costs directly, others have found spending on injuries in Nouna HDSS to be higher than other conditions such as malaria and chronic disease [71]. For conditions such as injury, which are unpredictable and high cost, community insurance schemes have been mooted as a way of limiting catastrophic expenditure[72]. Attempts to introduce such schemes in Nouna have suffered high dropout rates, possibly driven by fears around high cost and poor quality of health services[73]. There is evidence from Nouna that individuals enrolled on health insurance schemes received poorer quality healthcare services [74]. Such schemes, limited by low enrolment and selection bias, have failed to make a difference to overall population health including mortality [75 76].

Limitations:
This study has several limitations. CHAS injury data was self-reported and injuries were not independently verified. Nor was injury severity assessed or a clear definition of injury or disability provided. No minimum severity or clear definition can lead to inclusion of trivial injuries, overestimation of burden, and a lack of comparability across studies.

The time of injury was not reported. Recall loss is well established for community injury household surveys, particularly with respect of less severe injuries (causing less than 30 days disability)[48 77 78]. Some studies therefore use shorter recall time frames for their survey [50], or extrapolate minor injuries from the last 30 days reported incidence to calculate annual incidence for minor injuries [58]. Observing, confirming or correcting for recall was not possible in this study and we therefore may have underestimated the true incidence. As some injuries are known to be seasonal in Burkina Faso, this known recall bias may mean such injuries could have been misrepresented [8].

There were two injury cohorts described in this study, one reporting annual injury incidence and characteristics, the other reporting last health care visit for treatment following injury. This limited interpretation about health seeking behaviour for all respondents reporting recent injury.

Intentional injuries and domestic violence were not specifically differentiated from being struck or cut. Similarly road traffic was not a separate category, limited to falling from a motorcycle or

For peer review only - http://bmjopen.bmj.com/site/about/guidelines.xhtml

bicycle, perhaps justified by the low development status of rural Nouna. As RTCs are the main cause for Urban referral facility trauma care in Burkina Faso [9,10] and the only injury related SDG (SDG 3.6)[79] distinguishing these categories could allow future studies to compare within and across countries to inform preventative lessons and strategies.

This study was limited to a rural population. Urban and rural populations in sub-Saharan Africa experience differing burdens of injury [48,51]. Future urban comparisons could add perspective to inform national preventative and research strategies. This study was also confined to older adults and the injury burned for children and young adults is unknown. Fatal injuries were also not captured.

To build on these findings future research focussed on injuries could include fatal injuries, across the full population, with rural and urban comparison, capturing the time of injury relative to interview, with specific definitions for injury, disability and mechanism of injury categories, and matched to health system utilisation. This would help further understand the burden in Burkina Faso to inform preventative lessons and strategies as well as plan health system response. Nevertheless this study adds valuable insight into a relatively under researched topic in a country where little about injury burden or health care experience is known.

Conclusion:
This study has demonstrated the importance of injury burden in this older adult rural LMIC population contributing to the limited available published literature on this subject. Further research could inform preventative strategies including safer farming methods and the role of road traffic collisions, enable better understanding the association between adverse mental health and injury in this population, and strengthen health system readiness to provide quality care.

For peer review only

Table 1 Demographic information and disease states for those injured or not in preceding 12 months, and for those with or without a disability following injury.

Variable and statistical test for comparisons		All %<br>Sample size= 3028, apart from age and marital status (both N=3026), WHO DAS (N=3027) and frailty (N=2806)	Not injured in past 12months (%)<br>Sample size=2796, apart from age, marital status (both N=2794), WHO DAS (N=2795) and frailty (N=2607)	Injured in past 12months (%)<br>Sample size=232, apart from frailty (N=199)	Injured in past 12 months – No disability (%)<br>Sample size=127, apart from frailty (N=108)	Injured in past 12 months and suffered a disability (%)<br>Sample size=105, apart from frailty (N=91)
Median age (years) (IQR)		52 (45 – 62)	52 (45 – 62)	50 (45 – 60)	49 (44 – 61)	51 (45 – 58.5)
Female Sex (95% CI)		50.3 (48.6 - 52.1)	51.5 (49.7 – 53.4)	36.2 (30.3 – 42.6)	40.9 (32.8 – 49.6)	30.5 (22.5 – 39.8)
Marital status (95% CI)	Married/cohabiting	75.7 (74.1 – 77.2)	75.4 (73.8 – 77.0)	78.4 (72.7 – 83.3)	73.2 (64.9 – 80.2)	84.8 (76.7 – 90.4)
	Other	24.3 (22.8 – 25.9)	24.5 (23.0 – 26.2)	21.6 (16.8 – 27.3)	25.2 (19.8 – 35.1)	15.2 (9.6 – 23.3)
Socio-Economic Status	Quintile 1	19.9	20.2	16.4	17.3	15.2
	Quintile 2	19.8	20.1	15.9	19.7	11.4
	Quintile 3	20.0	19.8	22.4	19.7	25.7
	Quintile 4	20.3	20.2	20.7	17.3	24.8
	Quintile 5	20.0	19.6	24.6	26.0	22.9
What is the highest level of education you have completed? (95% CI)	No Formal Schooling	84.4 (83.1 – 85.7)	84.9 (83.5 – 86.2)	78.4 (72.7 – 83.3)	72.4 (64.1 – 79.5)	85.7 (77.8 – 91.2)
	Any schooling	15.6 (14.3 – 16.9)	15.1 (13.8 – 16.5)	21.6 (16.8 – 27.3)	27.6 (20.5 – 35.9)	14.3 (8.9 – 22.2)
PHQ-9 depression score (95% CI)	Normal or mild	91.9 (90.9 – 92.9)	92.0 (91.0 – 93.0)	91.0 (86.6 – 94.0)	90.6 (84.2 – 94.5)	90.5 (84.5 – 95.4)
	Moderate – severe*	8.1 (7.1 – 9.1)	8.0 (7.0 – 9.0)	9.0 (6.0 – 13.4)	9.4 (5.5 – 15.8)	8.5 (4.6 – 15.5)
Normalised WHO Disability Assessment Schedule 2.0 (median score IQR)		8.3 (2.1 – 22.9)	8.3 (2.1 – 22.9)	8.3 (2.1 – 25)	8.3 (2.1 – 22.9)	10.4 (2.1 – 27.1)
Normalised WHO Quality of Life (median and IQR)		59.4 (46.9 – 65.6)	59.4 (46.9 – 59.4)	56.3 (43.8 – 56.3)	56.3 (46.9 – 62.5)	56.3 (40.6 – 65.6)
Generalised Anxiety Disorder score (95% CI)	Abnormal	11.6 (10.5 – 12.8)	10.6 (9.5 – 11.8)	23.3 (18.3 – 29.1)	22.0 (15.7 – 30.0)	24.8 (17.5 – 33.8)
	Normal	88.4 (87.3 – 89.5)	89.4 (88.2 – 90.5)	76.7 (70.9 – 81.7)	78.0 (70.7 – 84.3)	75.2 (66.2 – 82.5)
Fried frailty score (95% CI)	Robust	45.3 (43.4 – 47.1)	45.3 (43.4 – 47.2)	44.7 (38.0 – 51.7)	38.0 (29.4 – 47.4)	52.7 (42.6 – 62.7)
	Pre-frail / Frail	54.7 (52.9 – 56.6)	54.7 (52.8 – 56.6)	55.3 (48.3 – 62.0)	62.0 (52.6 – 70.6)	47.3 (37.3 – 57.4)
*Score of 10 or more						

For peer review only

Table 2 - Logistic regression model for dependent variable of injury in last 12 months

		Odds Ratio	95% CI	P
Age		0.973	0.956 - 0.991	0.003
Sex	Male (ref)			
	Female	0.436	0.310 - 0.613	<0.001
Marital status	Married or cohabiting (ref)			
	Not married or cohabiting	1.247	0.812 - 1.917	0.313
Wealth Quintile	Quintile 1 (ref)			
	Quintile 2	1.011	0.597 - 1.712	0.967
	Quintile 3	1.453	0.886 - 2.382	0.138
	Quintile 4	1.367	0.823 - 2.272	0.227
	Quintile 5	1.795	1.079 - 2.985	0.024
Educational level	No Formal Schooling (ref)			
	Any Schooling	1.107	0.749 - 1.636	0.609
PHQ9	Normal or Mild (ref)			
	Moderate or Severe	0.559	0.295 - 1.059	0.075
GAD	Normal (ref)			
	abnormal	2.921	1.963 - 4.347	<0.001
Normalized WHO QOL score		0.981	0.97 - 0.993	0.002
Normalized WHO DAS 2.0 score (0-100)		1.004	0.992 - 1.016	0.512
Frailty	Robust (ref)			
	Pre-frail / Frail	0.967	0.704 - 1.327	0.834
**N = 2803**				

For peer review only

Table 3 - Multivariable logistic regression model for dependent variable of disability following injury in last 12 months including mechanism of injury

		Odds Ratio	95% CI	P
Age		1.016	0.977 – 1.057	0.426
Sex	Male (ref)			
	Female	0.471	0.215 – 1.033	0.06
Marital status	Married or cohabiting (ref)			
	Not married or cohabiting	0.433	0.15 – 1.25	0.122
Wealth Quintile	Quintile 1 (ref)			
	Quintile 2	0.636	0.187 – 2.167	0.469
	Quintile 3	1.09	0.368 – 3.226	0.876
	Quintile 4	2.05	0.666 – 6.306	0.211
	Quintile 5	1.521	0.503 – 4.596	0.457
Educational level	No Formal Schooling (ref)			
	Any Schooling	0.407	0.166 – 0.997	0.049
PHQ9	Normal or Mild (ref)			
	Moderate or Severe	0.426	0.107 – 1.689	0.225
GAD	Normal (ref)			
	abnormal	0.823	0.36 – 1.882	0.644
Normalized WHO QOL score		1.012	0.985 – 1.039	0.387
Normalized WHO DAS 2.0 score (0-100)		1.023	0.997 – 1.05	0.084
Frailty	Robust (ref)			
	Pre-frail / Frail	0.562	0.284 – 1.112	0.098
Mechanism of injury –	struck or hit by object (ref)			
	Fall	6.4	1.896 – 21.602	0.003
	Fall from motorbike	3.335	1.429 – 7.78	0.005
	Cut or stabbed	2.426	0.949 – 6.201	0.064
	Other	10.755	3.471 – 33.323	<0.001
**N = 199**				

For peer review only - http://bmjopen.bmj.com/site/about/guidelines.xhtml

Figure legends

Figure 1 – Mechanism of injury according to sex and associated disability

Figure 2 - Opinion and experience of healthcare received by those seeking care following an injury and those seeking care for another reason at last visit.

For peer review only

For peer review only - http://bmjopen.bmj.com/site/about/guidelines.xhtml

References:

1. Haagsma JA, Graetz N, Bolliger I, et al. The global burden of injury: incidence, mortality, disability-adjusted life years and time trends from the Global Burden of Disease study 2013. *Injury prevention : journal of the International Society for Child and Adolescent Injury Prevention* 2016;22(1):3-18. doi: 10.1136/injuryprev-2015-041616 [published Online First: 2015/12/05]
2. Gosselin RA, Spiegel DA, Coughlin R, et al. Injuries: the neglected burden in developing countries. *Bull World Health Organ* 2009;87(4):246-46a. [published Online First: 2009/06/25]
3. Bank. W. The world bank in Burkina Faso 2019 [Available from: http://www.worldbank.org/en/country/burkinafaso/overview
4. Global Health Data Exchange: Global Burden of Disease Results Tool [Available from: http://ghdx.healthdata.org/gbd-results-tool accessed 06/08/2020.
5. Mc Sween-Cadieux E, Dagenais C, Ridde V. A deliberative dialogue as a knowledge translation strategy on road traffic injuries in Burkina Faso: a mixed-method evaluation. *Health Res Policy Syst* 2018;16(1):113. doi: 10.1186/s12961-018-0388-8 [published Online First: 2018/11/22]
6. Ouedraogo R, Konsem T, Gyebre Y, et al. Experience with head and neck missile injuries at the yalgado university teaching hospital, ouagadougou, burkina faso. *J West Afr Coll Surg* 2012;2(4):1-9. [published Online First: 2012/10/01]
7. Sawadogo N, Garanet F, Traoré D, et al. [Morbidity of adolescents at the Kaya Regional Hospital Center (Burkina Faso)]. *Mali Med* 2018;33(1):29-34. [published Online First: 2018/11/30]
8. Dakouré PW, Diallo M, Traoré AC, et al. Trauma related to falls from trees treated in a specialized trauma centre in Burkina-Faso-one hundred and six cases treated in one year. *Int Orthop* 2015;39(12):2451-6. doi: 10.1007/s00264-015-2966-5 [published Online First: 2015/08/25]
9. Konsem T, Millogo M, Ouedraogo YI, et al. [Epidemiology of maxillo-facial traumatism s sequels at stomatology and maxillo-facial surgery service of Yalgado Ouedraogo University Hospital Center]. *Odontostomatol Trop* 2016;39(156):66-72. [published Online First: 2016/12/01]
10. Kabore AF, Ouedraogo A, Ki KB, et al. Head Computed Tomography Scan in Isolated Traumatic Brain Injury in a Low-Income Country. *World Neurosurg* 2017;107:382-88. doi: 10.1016/j.wneu.2017.07.160 [published Online First: 2017/08/23]
11. (WHO). WHO. Global status report on road safety 2018., 2018.
12. Bonnet E FA, Nikiema A, Ouedraogo M, Lechat L, Ridde V. . Se protéger en circulation réduit la gravité des blessures., 2016.
13. Soura AB, Lankoande B, Millogo R, et al. Comparing causes of death between formal and informal neighborhoods in urban Africa: evidence from Ouagadougou Health and Demographic Surveillance System. *Global health action* 2014;7:25523. doi: 10.3402/gha.v7.25523 [published Online First: 2014/11/08]
14. Streatfield PK, Khan WA, Bhuiya A, et al. Mortality from external causes in Africa and Asia: evidence from INDEPTH Health and Demographic

For peer review only - http://bmjopen.bmj.com/site/about/guidelines.xhtml

Surveillance System Sites. *Global health action* 2014;7:25366. doi: 10.3402/gha.v7.25366 [published Online First: 2014/11/08]

15. Gathecha GK, Ngaruiya C, Mwai W, et al. Prevalence and predictors of injuries in Kenya: findings from the national STEPs survey. *BMC public health* 2018;18(Suppl 3):1222-22. doi: 10.1186/s12889-018-6061-x
16. Wiseman T, Foster K, Curtis K. Mental health following traumatic physical injury: An integrative literature review. *Injury* 2013;44(11):1383-90. doi: 10.1016/j.injury.2012.02.015
17. Zatzick D, Roy-Byrne P, Russo J, et al. A randomized effectiveness trial of stepped collaborative care for acutely injured trauma survivors. *Arch Gen Psychiatry* 2004;61(5):498-506. doi: 10.1001/archpsyc.61.5.498 [published Online First: 2004/05/05]
18. Al-Sari UA, Tobias J, Clark E. Health-related quality of life in older people with osteoporotic vertebral fractures: a systematic review and meta-analysis. *Osteoporos Int* 2016;27(10):2891-900. doi: 10.1007/s00198-016-3648-x [published Online First: 2016/06/06]
19. Peeters CM, Visser E, Van de Ree CL, et al. Quality of life after hip fracture in the elderly: A systematic literature review. *Injury* 2016;47(7):1369-82. doi: 10.1016/j.injury.2016.04.018 [published Online First: 2016/05/15]
20. Schecter WP, Klassen C, O'Connor P, et al. Suicide: the unmet challenge of the trauma system. *Archives of surgery (Chicago, Ill : 1960)* 2005;140(9):902-4. doi: 10.1001/archsurg.140.9.902 [published Online First: 2005/09/24]
21. Zatzick DF, Kang SM, Kim SY, et al. Patients with recognized psychiatric disorders in trauma surgery: incidence, inpatient length of stay, and cost. *J Trauma* 2000;49(3):487-95. doi: 10.1097/00005373-200009000-00017 [published Online First: 2000/09/26]
22. Cange CW, Wirtz AL, Ky-Zerbo O, et al. Effects of traumatic events on sex workers' mental health and suicide intentions in Burkina Faso: a trauma-informed approach. *Sex Health* 2019;16(4):348-57. doi: 10.1071/sh17213 [published Online First: 2019/07/12]
23. Ismayilova L, Gaveras E, Blum A, et al. Maltreatment and Mental Health Outcomes among Ultra-Poor Children in Burkina Faso: A Latent Class Analysis. *PloS one* 2016;11(10):e0164790. doi: 10.1371/journal.pone.0164790 [published Online First: 2016/10/21]
24. Mock C, Joshipura M, Arreola-Risa C, et al. An estimate of the number of lives that could be saved through improvements in trauma care globally. *World J Surg* 2012;36(5):959-63. doi: 10.1007/s00268-012-1459-6 [published Online First: 2012/03/16]
25. Darby C VN, Murray CJ, De Silva A. World Health Organization (WHO): Strategy on measuring responsiveness, 2003.
26. Whitaker J, Nepogodiev D, Leather A, et al. Assessing barriers to quality trauma care in low and middle-income countries: A Delphi study. *Injury* 2019 doi: 10.1016/j.injury.2019.12.035 [published Online First: 2019/12/31]
27. Odland ML, Whitaker J, Nepogodiev D, et al. Identifying, Prioritizing and Visually Mapping Barriers to Injury Care in Rwanda: A Multi-disciplinary Stakeholder Exercise. *World Journal of Surgery* 2020 doi: 10.1007/s00268-020-05571-6

For peer review only

28. Kruk ME, Gage AD, Arsenault C, et al. High-quality health systems in the Sustainable Development Goals era: time for a revolution. *Lancet Glob Health* 2018 doi: 10.1016/s2214-109x(18)30386-3 [published Online First: 2018/09/10]
29. von Elm E, Altman DG, Egger M, et al. The Strengthening the Reporting of Observational Studies in Epidemiology (STROBE) statement: guidelines for reporting observational studies. *J Clin Epidemiol* 2008;61(4):344-9. doi: 10.1016/j.jclinepi.2007.11.008 [published Online First: 2008/03/04]
30. Sie A, Louis VR, Gbangou A, et al. The Health and Demographic Surveillance System (HDSS) in Nouna, Burkina Faso, 1993-2007. *Glob Health Action* 2010;3 doi: 10.3402/gha.v3i0.5284 [published Online First: 2010/09/18]
31. Odhiambo Sewe M, Bunker A, Ingole V, et al. Estimated Effect of Temperature on Years of Life Lost: A Retrospective Time-Series Study of Low-, Middle-, and High-Income Regions. *Environ Health Perspect* 2018;126(1):017004-04. doi: 10.1289/EHP1745
32. Witham MD, Davies JI, Bärnighausen T, et al. Frailty and physical performance in the context of extreme poverty: a population-based study of older adults in rural Burkina Faso. *Wellcome Open Res* 2019;4:135-35. doi: 10.12688/wellcomeopenres.15455.1
33. Odland ML, Payne C, Witham MD, et al. Epidemiology of multimorbidity in conditions of extreme poverty: a population-based study of older adults in rural Burkina Faso. *BMJ global health* 2020;5(3):e002096-e96. doi: 10.1136/bmjgh-2019-002096
34. Hartung C. Open Data Kit: Tools to Build Information Services for Developing Regions. 2010
35. Ratcliffe HL, Bell G, Awoonor-Williams K, et al. Towards patient-centred care in Ghana: health system responsiveness, self-rated health and experiential quality in a nationally representative survey. *BMJ Open Qual* 2020;9(2):e000886. doi: 10.1136/bmjoq-2019-000886
36. Baltussen RM, Yé Y, Haddad S, et al. Perceived quality of care of primary health care services in Burkina Faso. *Health Policy Plan* 2002;17(1):42-8. doi: 10.1093/heapol/17.1.42 [published Online First: 2002/02/28]
37. Miller JS, Mhalu A, Chalamilla G, et al. Patient satisfaction with HIV/AIDS care at private clinics in Dar es Salaam, Tanzania. *AIDS care* 2014;26(9):1150-4. doi: 10.1080/09540121.2014.882487 [published Online First: 2014/02/07]
38. Plummer F, Manea L, Trepel D, et al. Screening for anxiety disorders with the GAD-7 and GAD-2: a systematic review and diagnostic metaanalysis. *Gen Hosp Psychiatry* 2016;39:24-31. doi: 10.1016/j.genhosppsych.2015.11.005 [published Online First: 2016/01/01]
39. Spitzer RL, Kroenke K, Williams JB. Validation and utility of a self-report version of PRIME-MD: the PHQ primary care study. Primary Care Evaluation of Mental Disorders. Patient Health Questionnaire. *JAMA* 1999;282(18):1737-44. doi: 10.1001/jama.282.18.1737 [published Online First: 1999/11/24]
40. Kroenke K, Spitzer RL, Williams JB. The PHQ-9: validity of a brief depression severity measure. *J Gen Intern Med* 2001;16(9):606-13. doi:

For peer review only - http://bmjopen.bmj.com/site/about/guidelines.xhtml

10.1046/j.1525-1497.2001.016009606.x [published Online First: 2001/09/15]
41. Schmidt S, Muhlan H, Power M. The EUROHIS-QOL 8-item index: psychometric results of a cross-cultural field study. *Eur J Public Health* 2006;16(4):420-8. doi: 10.1093/eurpub/cki155 [published Online First: 2005/09/06]
42. Gomez-Olive FX, Schroders J, Aboderin I, et al. Variations in disability and quality of life with age and sex between eight lower income and middle-income countries: data from the INDEPTH WHO-SAGE collaboration. *BMJ Glob Health* 2017;2(4):e000508. doi: 10.1136/bmjgh-2017-000508 [published Online First: 2018/01/16]
43. Garin O, Ayuso-Mateos JL, Almansa J, et al. Validation of the "World Health Organization Disability Assessment Schedule, WHODAS-2" in patients with chronic diseases. *Health Qual Life Outcomes* 2010;8:51. doi: 10.1186/1477-7525-8-51 [published Online First: 2010/05/21]
44. Fried LP, Tangen CM, Walston J, et al. Frailty in older adults: evidence for a phenotype. *J Gerontol A Biol Sci Med Sci* 2001;56(3):M146-56. doi: 10.1093/gerona/56.3.m146 [published Online First: 2001/03/17]
45. Witham MDD, J.I. Barning, T. Boun, M. Manne-Goi. Frailty and physical performance in the context of extreme poverty: a population-based study of older adults in rural Burkina Faso. *Wellcome Open Res* 2019
46. Filmer D, Pritchett LH. Estimating wealth effects without expenditure data--or tears: an application to educational enrollments in states of India. *Demography* 2001;38(1):115-32. doi: 10.1353/dem.2001.0003 [published Online First: 2001/03/03]
47. Wickham H. ggplot2: Elegant Graphics for Data Analysis: Springer-Verlag New York 2009.
48. Moshiro C, Heuch I, Åstrøm AN, et al. Injury morbidity in an urban and a rural area in Tanzania: an epidemiological survey. *BMC Public Health* 2005;5(1):11. doi: 10.1186/1471-2458-5-11
49. Petroze RT, Joharifard S, Groen RS, et al. Injury, disability and access to care in Rwanda: results of a nationwide cross-sectional population study. *World J Surg* 2015;39(1):62-9. doi: 10.1007/s00268-014-2544-9 [published Online First: 2014/05/29]
50. Olawale OA, Owoaje ET. Incidence and pattern of injuries among residents of a rural area in South-Western Nigeria: a community-based study. *BMC Public Health* 2007;7(1):246. doi: 10.1186/1471-2458-7-246
51. Tayeb SE, Abdalla S, Mørkve O, et al. Injuries in Khartoum state, the Sudan: a household survey of incidence and risk factors. *International Journal of Injury Control and Safety Promotion* 2014;21(2):144-53. doi: 10.1080/17457300.2013.792283
52. Stewart K-AA, Groen RS, Kamara TB, et al. Traumatic injuries in developing countries: report from a nationwide cross-sectional survey of Sierra Leone. *JAMA surgery* 2013;148(5):463-69. doi: 10.1001/jamasurg.2013.1341
53. Sorenson SB. Gender disparities in injury mortality: consistent, persistent, and larger than you'd think. *Am J Public Health* 2011;101 Suppl 1(Suppl 1):S353-S58. doi: 10.2105/AJPH.2010.300029 [published Online First: 2011/07/21]

For peer review only

54. Nantulya VM, Reich MR. Equity dimensions of road traffic injuries in low- and middle-income countries. *Inj Control Saf Promot* 2003;10(1-2):13-20. doi: 10.1076/icsp.10.1.13.14116 [published Online First: 2003/05/30]
55. (WHO). WHO. Injuries and Violence: The Facts., 2014.
56. Wyatt GE, Thames A, Simbayi L, et al. Trauma and mental health in South Africa: Overview. *Psychol Trauma* 2017;9(3):249-51. doi: 10.1037/tra0000144
57. El Tayeb S, Abdalla S, Heuch I, et al. Socioeconomic and disability consequences of injuries in the Sudan: a community-based survey in Khartoum State. *Injury prevention : journal of the International Society for Child and Adolescent Injury Prevention* 2015;21(e1):e56-62. doi: 10.1136/injuryprev-2013-040818 [published Online First: 2013/11/15]
58. Mock CN, Abantanga F, Cummings P, et al. Incidence and outcome of injury in Ghana: a community-based survey. *Bull World Health Organ* 1999;77(12):955-64. [published Online First: 2000/02/19]
59. Kuzma K, Lim AG, Kepha B, et al. The Tanzanian trauma patients' prehospital experience: a qualitative interview-based study. *BMJ Open* 2015;5(4) doi: 10.1136/bmjopen-2014-006921
60. Chokotho L, Jacobsen KH, Burgess D, et al. A review of existing trauma and musculoskeletal impairment (TMSI) care capacity in East, Central, and Southern Africa. *Injury* 2016;47(9):1990-5. doi: 10.1016/j.injury.2015.10.036 [published Online First: 2016/05/15]
61. Stewart BT, Quansah R, Gyedu A, et al. Strategic Assessment of Trauma Care Capacity in Ghana. *World J Surg* 2015;39(10):2428-40. doi: 10.1007/s00268-015-3132-3 [published Online First: 2015/07/15]
62. Mock C LJ, Goosen J, Joshipura M, Peden M. Guidelines for essential trauma care. Geneva, Switzerland: World Health Organization, 2004.
63. Burke TF, Hines R, Ahn R, et al. Emergency and urgent care capacity in a resource-limited setting: an assessment of health facilities in western Kenya. *BMJ Open* 2014;4(9):e006132. doi: 10.1136/bmjopen-2014-006132
64. Mulwafu W, Chokotho L, Mkandawire N, et al. Trauma care in Malawi: A call to action. *Malawi Medical Journal* 2017;29(2):198-202.
65. Banuri S, de Walque D, Keefer P, et al. The use of video vignettes to measure health worker knowledge. Evidence from Burkina Faso. *Soc Sci Med* 2018;213:173-80. doi: 10.1016/j.socscimed.2018.07.046 [published Online First: 2018/08/12]
66. Duysburgh E, Temmerman M, Yé M, et al. Quality of antenatal and childbirth care in rural health facilities in Burkina Faso, Ghana and Tanzania: an intervention study. *Tropical medicine & international health : TM & IH* 2016;21(1):70-83. doi: 10.1111/tmi.12627 [published Online First: 2015/10/28]
67. Beogo I, Huang N, Gagnon MP, et al. Out-of-pocket expenditure and its determinants in the context of private healthcare sector expansion in sub-Saharan Africa urban cities: evidence from household survey in Ouagadougou, Burkina Faso. *BMC research notes* 2016;9:34. doi: 10.1186/s13104-016-1846-4 [published Online First: 2016/01/23]
68. Wesson HKH, Boikhutso N, Bachani AM, et al. The cost of injury and trauma care in low- and middle-income countries: a review of economic evidence.

For peer review only - http://bmjopen.bmj.com/site/about/guidelines.xhtml

Health policy and planning 2014;29(6):795-808. doi: 10.1093/heapol/czt064 [published Online First: 2013/10/05]
69. Kotagal M, Agarwal-Harding KJ, Mock C, et al. Health and economic benefits of improved injury prevention and trauma care worldwide. *PloS one* 2014;9(3):e91862. doi: 10.1371/journal.pone.0091862 [published Online First: 2014/03/15]
70. Wu Y, Zhang W, Zhang L, et al. Non-fatal injuries treated outside a hospital in Hunan, China: results from a household interview survey. *European journal of public health* 2016;27(2):331-34. doi: 10.1093/eurpub/ckw114
71. Su TT, Pokhrel S, Gbangou A, et al. Determinants of household health expenditure on western institutional health care. *Eur J Health Econ* 2006;7(3):199-207. doi: 10.1007/s10198-006-0354-1 [published Online First: 2006/05/05]
72. Meara JG, Leather AJ, Hagander L, et al. Global Surgery 2030: evidence and solutions for achieving health, welfare, and economic development. *Lancet (London, England)* 2015;386(9993):569-624. doi: 10.1016/s0140-6736(15)60160-x [published Online First: 2015/05/01]
73. Dong H, De Allegri M, Gnawali D, et al. Drop-out analysis of community-based health insurance membership at Nouna, Burkina Faso. *Health Policy* 2009;92(2-3):174-9. doi: 10.1016/j.healthpol.2009.03.013 [published Online First: 2009/04/28]
74. Robyn PJ, Fink G, Sié A, et al. Health insurance and health-seeking behavior: evidence from a randomized community-based insurance rollout in rural Burkina Faso. *Soc Sci Med* 2012;75(4):595-603. doi: 10.1016/j.socscimed.2011.12.018 [published Online First: 2012/02/11]
75. Hounton S, Byass P, Kouyate B. Assessing effectiveness of a community based health insurance in rural Burkina Faso. *BMC Health Serv Res* 2012;12:363. doi: 10.1186/1472-6963-12-363 [published Online First: 2012/10/23]
76. Fink G, Robyn PJ, Sié A, et al. Does health insurance improve health?: Evidence from a randomized community-based insurance rollout in rural Burkina Faso. *J Health Econ* 2013;32(6):1043-56. doi: 10.1016/j.jhealeco.2013.08.003 [published Online First: 2013/10/10]
77. Mock C, Acheampong F, Adjei S, et al. The effect of recall on estimation of incidence rates for injury in Ghana. *International journal of epidemiology* 1999;28(4):750-5. [published Online First: 1999/09/10]
78. Moshiro C, Heuch I, Astrøm AN, et al. Effect of recall on estimation of non-fatal injury rates: a community based study in Tanzania. *Injury prevention : journal of the International Society for Child and Adolescent Injury Prevention* 2005;11(1):48-52. doi: 10.1136/ip.2004.005645
79. World Health Organization. Sustainable Development Goal 3: Health 2015 [Available from: https://www.who.int/topics/sustainable-development-goals/targets/en/.

For peer review only - http://bmjopen.bmj.com/site/about/guidelines.xhtml

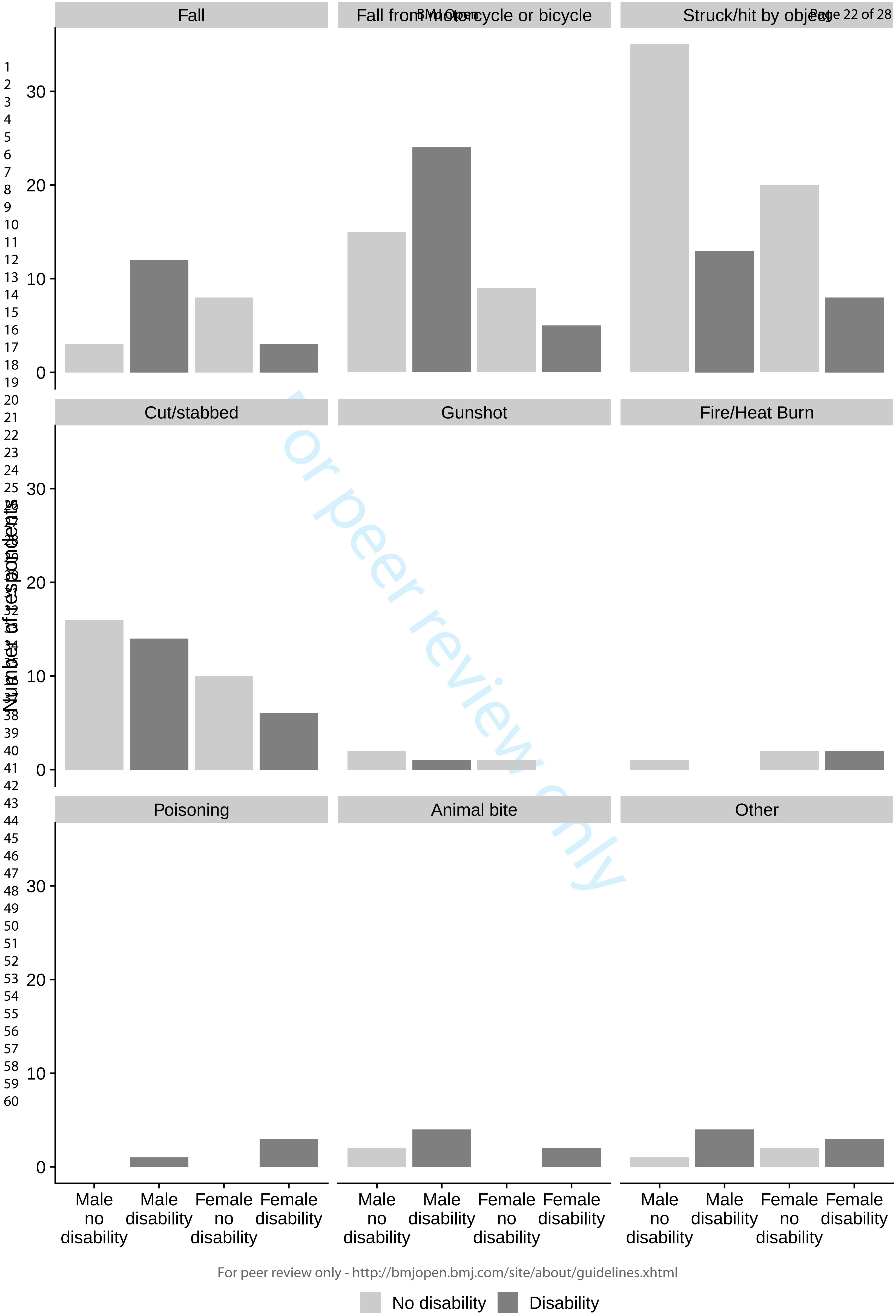

For peer review only

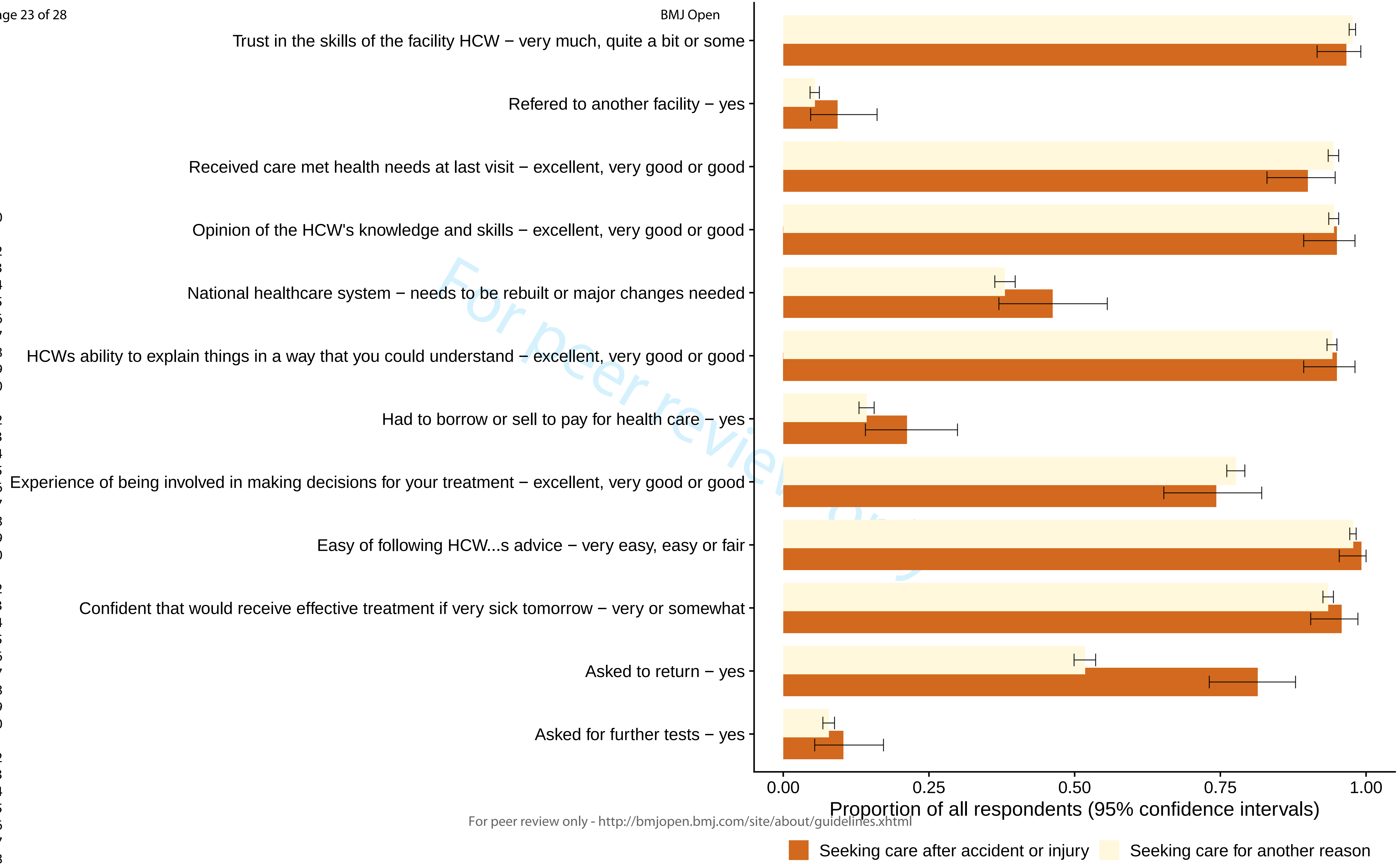

For peer review only - http://bmjopen.bmj.com/site/about/guidelines.xhtml

Appendices

Appendix table 1 - Mechanism of injury according to wealth quintile, for all those injured in past 12 months with number of males in brackets.

Wealth Quintile	Fall N (n of males)	Fall from motorcycle or bicycle N (n of males)	Struck / hit by object N (n of males)	Cut / stabbed N (n of males)	Gunshot N (n of males)	Fire / Heat Burn N (n of males)	Poisoning N (n of males)	Animal bite N (n of males)	Other N (n of males)
1	6 (4)	3 (3)	10 (7)	11 (5)	1 (1)	1 (0)	0 (0)	2 (1)	4 (2)
2	5 (1)	6 (5)	17 (13)	2 (1)	2 (2)	1 (1)	3 (0)	1 (1)	0 (0)
3	9 (5)	12 (6)	12 (8)	15 (11)	0 (0)	0 (0)	1 (1)	0 (0)	3 (2)
4	3 (3)	13 (10)	20 (11)	5 (5)	0 (0)	2 (0)	0 (0)	2 (2)	3 (1)
5	3 (2)	19 (15)	17 (9)	13 (8)	1 (0)	1 (0)	0 (0)	3 (2)	0 (0)
Total	26 (15)	53 (39)	76 (48)	46 (30)	4 (3)	5 (1)	4 (1)	8 (6)	10 (5)

Appendix table 2 - Binary logistic regression for falling from a motorcycle or bicycle and wealth quintile.

	Odds Ratio	95% C.I.	P Value
Quintile 1 (ref)			
Quintile 2	2.26	0.52 - 9.80	0.277
Quintile 3	3.50	0.91 - 13.42	0.068
Quintile 4	4.33	1.14 - 16.55	0.032
Quintile 5	5.83	1.59 - 21.43	0.008

Appendix table 3 Opinion and experience of healthcare received by those seeking care following an injury and those seeking care for another reason at last visit.

	Seeking care after an accident or injury % (95% CI) Number responders (N) = 119 unless otherwise stated	Seeking care for another reason n % (95% CI) Number responders (N) = 2882 unless otherwise stated
**Opinion of care received**		
Confident that would receive effective treatment if very sick tomorrow		
Very or somewhat confident	95.8 (90.5 – 98.2)	93.5 (92.6 – 94.4)
Not very or not at all confident	4.2 (1.8 – 9.5)	6.5 (5.6 – 7.4)
Overall view of national healthcare system		
Needs to be rebuilt or major changes needed	46.2 (37.5 – 55.2)	38.0 (36.3 – 39.8) (N=2909)
Only minor changes needed	53.8 (44.9 – 62.5)	62.0 (60.2 – 63.7) (N=2909)
Overall how well did received care meet health needs at last visit?		
Excellent, very good or good	90.0 (83.2 – 94.1)	94.4 (93.5 – 95.2)
Fair or poor	10.0 (5.9 – 16.8)	5.5 (4.8 – 6.5)
Do you trust in the skills and abilities of the HCW at the facility?		
Very much, quite a bit or some.	96.6 (91.7 – 98.7)	97.7 (97.1 – 98.2) (N=2879)
Very little or not at all	3.4 (1.3 – 8.3)	2.3 (1.8 – 2.9) (N=2879)
What is your opinion of the care provider's knowledge and skills?		
Excellent, very good or good	95.0 (89.4 – 97.7)	94.5 (93.3 – 95.0) (N=2873)
Fair or poor	5.0 (2.3 – 10.6)	5.5 (5.0 – 6.7) (N=2873)
What do you think about the provider's ability to explain things in a way that you could understand?		

For peer review only - http://bmjopen.bmj.com/site/about/guidelines.xhtml

	Excellent, very good or good	95.0 (89.4 – 97.7)	94.2 (93.3 – 95.0) (N=2864)
	Fair or poor	5.0 (2.3 – 10.6)	5.8 (5.0 – 6.7) (N=2864)
How easy or difficult was it for you to follow the provider's advice?			
	Very Easy, easy or fair	99.2 (95.4 – 99.9)	97.8 (97.3 – 98.3) (N=2877)
	Hard or very hard	0.8 (0.2 – 4.6)	2.2 (1.7 – 2.8) (N=2877)
What do you think about your experience of being involved in making decisions for your treatment?			
	Excellent, very good or good	74.3 (65.6 – 81.5) (N=113)	77.7 (76.1 – 79.2) (N=2748)
	Fair or poor	25.7 (18.5 – 34.4) (N=113)	22.3 (20.8 – 23.9) (N=2748)
**Experience of care processes**			
Did care provider refer to another facility? - Yes		9.3 (5.2 – 15.8)	5.4 (4.6 – 6.3) (N=2872)
Did care provider ask for further tests? - Yes		10.3 (6.0 – 17.1) (N=117)	7.8 (6.9 – 8.8) (N=2859)
Did the care provider ask you to come back? - Yes		81.4 (72.7 – 86.8)	51.8 (50.0 – 53.6) (N=2881)
Had to borrow or sell to pay for health care episode? - Yes		21.2 (14.7 – 29.7) (N=113)	14.3 (13.0 – 15.6) (N=2881)
How long did you wait before your consultation (minutes)? (Median and IQR) (Mann-Witney U)		10 (5-15) (N=118)	20 (10-30) (N=2885)<br><br>P = 0.002
How much time did you spend with the care provider (minutes)? (Median and IQR) (Mann-Witney U)		20 (15-30) (N=118)	15 (20-25) (N=2885)<br><br>P = 0.002

For peer review only

Reporting checklist for cross sectional study.

Based on the STROBE cross sectional guidelines.

		Reporting Item	Page Number
**Title and abstract**			
Title	#1a	Indicate the study's design with a commonly used term in the title or the abstract	1
Abstract	#1b	Provide in the abstract an informative and balanced summary of what was done and what was found	2
**Introduction**			
Background / rationale	#2	Explain the scientific background and rationale for the investigation being reported	4
Objectives	#3	State specific objectives, including any prespecified hypotheses	4
**Methods**			
Study design	#4	Present key elements of study design early in the paper	5
Setting	#5	Describe the setting, locations, and relevant dates, including periods of recruitment, exposure, follow-up, and data collection	5
Eligibility criteria	#6a	Give the eligibility criteria, and the sources and methods of selection of participants.	5
	#7	Clearly define all outcomes, exposures, predictors, potential confounders, and effect modifiers. Give diagnostic criteria, if applicable	5-6
Data sources / measurement	#8	For each variable of interest give sources of data and details of methods of assessment (measurement). Describe comparability of assessment methods if there is more than one group. Give information separately for for exposed and unexposed groups if applicable.	5

For peer review only

Bias	#9	Describe any efforts to address potential sources of bias	5
Study size	#10	Explain how the study size was arrived at	5
Quantitative variables	#11	Explain how quantitative variables were handled in the analyses. If applicable, describe which groupings were chosen, and why	5-6
Statistical methods	#12a	Describe all statistical methods, including those used to control for confounding	6
Statistical methods	#12b	Describe any methods used to examine subgroups and interactions	6
Statistical methods	#12c	Explain how missing data were addressed	6
Statistical methods	#12d	If applicable, describe analytical methods taking account of sampling strategy	NA
Statistical methods	#12e	Describe any sensitivity analyses	6
**Results**			
Participants	#13a	Report numbers of individuals at each stage of study—eg numbers potentially eligible, examined for eligibility, confirmed eligible, included in the study, completing follow-up, and analysed. Give information separately for for exposed and unexposed groups if applicable.	7
Participants	#13b	Give reasons for non-participation at each stage	NA
Participants	#13c	Consider use of a flow diagram	NA
Descriptive data	#14a	Give characteristics of study participants (eg demographic, clinical, social) and information on exposures and potential confounders. Give information separately for exposed and unexposed groups if applicable.	7, 11
Descriptive data	#14b	Indicate number of participants with missing data for each variable of interest	11, 12, 13, Supplementary appendix tables p1&2

For peer review only

Outcome data	#15	Report numbers of outcome events or summary measures. Give information separately for exposed and unexposed groups if applicable.	7, 11, 12, 13, Supplementary appendix tables p1&2
Main results	#16a	Give unadjusted estimates and, if applicable, confounder-adjusted estimates and their precision (eg, 95% confidence interval). Make clear which confounders were adjusted for and why they were included	7, 11, 12, 13
Main results	#16b	Report category boundaries when continuous variables were categorized	5
Main results	#16c	If relevant, consider translating estimates of relative risk into absolute risk for a meaningful time period	NA
Other analyses	#17	Report other analyses done—e.g., analyses of subgroups and interactions, and sensitivity analyses	7
**Discussion**			
Key results	#18	Summarise key results with reference to study objectives	8, 9
Limitations	#19	Discuss limitations of the study, taking into account sources of potential bias or imprecision. Discuss both direction and magnitude of any potential bias.	9, 10
Interpretation	#20	Give a cautious overall interpretation considering objectives, limitations, multiplicity of analyses, results from similar studies, and other relevant evidence.	8-10
Generalisability	#21	Discuss the generalisability (external validity) of the study results	9-10
**Other Information**			
Funding	#22	Give the source of funding and the role of the funders for the present study and, if applicable, for the original study on which the present article is based	3

Notes:

For peer review only

The STROBE checklist is distributed under the terms of the Creative Commons Attribution License CC-BY.

This checklist was completed on 06. October 2020 using https://www.goodreports.org/, a tool made by the EQUATOR Network in collaboration with Penelope.ai

For peer review only

BMJ Open

Non-fatal injuries in rural Burkina Faso amongst older adults, disease burden and health system responsiveness, a cross-sectional household survey.

Journal:	*BMJ Open*
Manuscript ID	bmjopen-2020-045621.R1
Article Type:	Original research
Date Submitted by the Author:	08-Feb-2021
Complete List of Authors:	Whitaker, John; King's College London Faculty of Life Sciences and Medicine, Centre for Global Health and Health Partnerships; Royal Centre for Defence Medicine, Academic Department of Military Surgery and Trauma<br>Harling, Guy; University College London, Institute for Global Health; Harvard University T H Chan School of Public Health, Department of Epidemiology<br>Sie, Ali; Centre de Recherche en Sante de Nouna<br>Bountogo, Mamadou; Centre de Recherche en Sante de Nouna, Nouna; Université de Ouagadougou, Ouaga<br>Hirschhorn, Lisa; Northwestern University Feinberg School of Medicine, medical Social Sciences; Northwestern University Feinberg School of Medicine,<br>Manne-Goehler, Jennifer; Harvard Medical School, Division of Infectious Diseases, Massachusetts General Hospital<br>Bärnighausen, Till ; Heidelberg University, Institute of Public Health<br>Davies, Justine ; University of Birmingham Institute of Applied Health Research; University of the Witwatersrand School of Public Health, MRC/Wits Rural Public Health & Health Transitions Research Unit (Agincourt
**Primary Subject Heading**:	Epidemiology
Secondary Subject Heading:	Global health
Keywords:	Trauma management < ORTHOPAEDIC & TRAUMA SURGERY, HEALTH SERVICES ADMINISTRATION & MANAGEMENT, EPIDEMIOLOGY, TROPICAL MEDICINE

SCHOLARONE™
Manuscripts

I, the Submitting Author has the right to grant and does grant on behalf of all authors of the Work (as defined in the below author licence), an exclusive licence and/or a non-exclusive licence for contributions from authors who are: i) UK Crown employees; ii) where BMJ has agreed a CC-BY licence shall apply, and/or iii) in accordance with the terms applicable for US Federal Government officers or employees acting as part of their official duties; on a worldwide, perpetual, irrevocable, royalty-free basis to BMJ Publishing Group Ltd ("BMJ") its licensees and where the relevant Journal is co-owned by BMJ to the co-owners of the Journal, to publish the Work in this journal and any other BMJ products and to exploit all rights, as set out in our licence.

The Submitting Author accepts and understands that any supply made under these terms is made by BMJ to the Submitting Author unless you are acting as an employee on behalf of your employer or a postgraduate student of an affiliated institution which is paying any applicable article publishing charge ("APC") for Open Access articles. Where the Submitting Author wishes to make the Work available on an Open Access basis (and intends to pay the relevant APC), the terms of reuse of such Open Access shall be governed by a Creative Commons licence – details of these licences and which Creative Commons licence will apply to this Work are set out in our licence referred to above.

Other than as permitted in any relevant BMJ Author's Self Archiving Policies, I confirm this Work has not been accepted for publication elsewhere, is not being considered for publication elsewhere and does not duplicate material already published. I confirm all authors consent to publication of this Work and authorise the granting of this licence.

For peer review only

Title: **Non-fatal injuries in rural Burkina Faso amongst older adults, disease burden and health system responsiveness, a cross-sectional household survey.**

Authors

John Whitaker 1,2 – corresponding author
Guy Harling 3,4,5,6
Ali Sie 7
Mamadou Bountogo 7
Lisa R Hirschhorn 8
Jennifer Manne-Goehler 9
Till Bärnighausen 3, 10
Justine Davies 5, 11

Affiliations

1 - King's Centre for Global Health and Health Partnerships, School of Population Health & Environmental Sciences, Faculty of Life Sciences and Medicine, King's College London, London, UK. Room 2.13, Global Health Offices, Weston Education Centre, Cutcombe Road, London, SE5 9RJ, UK. john.k.whitaker@kcl.ac.uk.
2 - Academic Department of Military Surgery and Trauma, Royal Centre for Defence Medicine, Birmingham, UK.
3 - Africa Health Research Institute, KwaZulu-Natal, South Africa.
4 - Institute for Global Health, University College London, London.
5 - MRC/Wits Rural Public Health & Health Transitions Research Unit (Agincourt), University of the Witwatersrand, Johannesburg-Braamfontein, South Africa.
6 - Department of Epidemiology & Harvard Center for Population and Development Studies, Harvard T.H. Chan School of Public Health, Boston, MA, USA.
7 - Centre de Recherche en Sante de Nouna, Nouna, Burkina Faso.
8 - Department of Medical Social Sciences, Feinberg School of Medicine, Northwestern University, Chicago, IL, USA.
9 - Division of Infectious Diseases, Massachusetts General Hospital, Harvard Medical School, Boston, Massachusetts, USA.
10 - Heidelberg Institute of Global Health (HIGH), Faculty of Medicine and University Hospitals, University of Heidelberg, Heidelberg, Germany.
11 - Centre for Applied Health Research, University of Birmingham, Birmingham, UK.

For peer review only

Abstract:

Objectives: This study aimed to evaluate the epidemiology of injury as well as patient-reported health system responsiveness following injury and how this compares with non-injured patient experience, in older individuals in rural Burkina Faso.

Design: Cross sectional household survey. Secondary analysis of the CRSN Heidelberg Aging Study dataset (CHAS).

Setting: Rural Burkina Faso

Participants: 3028 adults, over 40, from multiple ethnic groups, were randomly sampled from the 2015 Nouna Health and Demographic Surveillance Site census.

Primary and secondary outcome measures: Primary outcome was incidence of injury. Secondary outcomes were incidence of injury related disability and patient reported health system responsiveness following injury.

Results: 7.7% (232/3028) of the population reported injury in the preceding 12 months. In multivariable analyses, younger age, male sex, highest wealth quintile, an abnormal Generalised Anxiety Disorder score, and lower Quality of Life score were all associated with injury. The most common mechanism of injury was being struck or hit by an object, 32.8%. In multivariable analysis, only education was significantly negatively associated with odds of disability (OR 0.407, 95%CI 0.17–0.997). Across all survey participants, 3.9% (119/3028) reported their most recent care seeking episode was following injury, rather than for another condition. Positive experience and satisfaction with care were reported following injury, with shorter median wait times (10 vs 20 minutes, P=0.002) and longer consultation times (20 vs 15 minutes P=0.002) than care for another reason. Injured patients were also asked to return to health facilities more often than those seeking care for another reason, 81.4% (95%CI 73.1%–87.9%) vs 54.8% (95%CI 49.9%–53.6%).

Conclusions: Injury is an important disease burden in this older adult rural LMIC population. Further research could inform preventative strategies, including safer rural farming methods, explore the association between adverse mental health and injury, and strengthen health system readiness to provide quality care.

Keywords
Wounds and injuries, developing countries, health services research, epidemiology, surveys and questionnaires.

Word count
4076

Article Summary

Strengths and limitations of this study

- Through a random sampling strategy, our household survey was able to establish the incidence of non-fatal injury in rural Burkina Faso, where little empirical data on injury exists.
- By including variables of psychological morbidity and quality of life, we were able to explore associations with those reporting injuries, an understudied aspect of injury burden on low-income settings.
- By establishing the most recent reason for accessing care, we were able to compare the health system responsiveness following injury with other conditions.
- The study was cross sectional, which limits the causal interpretation of our findings.
- The survey lacked clear definitions of injury and disability, which may have led to an overestimation of burden.

For peer review only

Author Contributions

TB, AS and GH conceived and designed the overall CSRN CHAS study.

MB and GH co-ordinated baseline data collection and preparation.

JD, JM-G and LRH contributed to the design of the CSRN CHAS household survey.

JW and JD designed the current study.

JW conducted the analysis, wrote and revised the manuscript.

JD supervised the analysis, write up and development of the manuscript.

All authors substantively reviewed manuscripts, inputted into revisions and approved the final manuscript.

Funding statement

Funding Support for the CRSN Heidelberg Aging Study and for TB was provided by the Alexander von Humboldt Foundation through the Alexander von Humboldt Professor award (no grant number exists) to Till Bärnighausen, funded by the German Federal Ministry of Education and Research. GH is supported by a fellowship (210479/Z/18/Z) from both the Wellcome Trust and Royal Society. JMG was supported by Grant Number T32 AI007433 from the National Institute of Allergy and Infectious Diseases. JW is a serving member of the UK Defence Medical Services.

Competing interests: - None declared.

Patient consent for publication: - Not required.

Ethics approval: - Ethical approval was obtained from Ethics Commission I of the medical faculty Heidelberg (S-120/2018), the Burkina Faso Comité d'Ethique pour la Recherche en Santé (CERS) in Ouagadougou (2018-4-045) and the Institutional Ethics Committee (CIE) of the CRSN (2018-04). Oral assent was sought from all village elders. Written informed consent was obtained from each participant, and a literate witness assisted in cases of illiteracy

Data availability statement: - Data may be obtained from a third party and are not publicly available. Data are not publicly available as consent was not given by participants for this to take place. This is in part because entire age cohorts of some villages are included in the data set, potentially allowing for deductive disclosure with sufficient local information. For this reason, anonymised data are available from CHAS study data controllers only following signature of a data use agreement restricting onward transmission. Anyone wishing to replicate the analyses presented or conduct further collaborative analyses using CHAS (which are welcomed and considered based on a letter of intent), should contact Dr Guy Harling (g.harling@ucl.ac.uk) in the first instance.

For peer review only

Introduction

Injury is a neglected but important cause of avoidable disability and causes more than five million deaths globally every year [1], more than tuberculosis, malaria and HIV combined. Low- and middle-Income Countries (LMICs) disproportionately bear this burden with 90% of global injury related deaths [2]. However, injury related deaths are only the tip of the iceberg with an estimated one billion people sustaining injuries that require healthcare annually [1].

Burkina Faso is a landlocked country of 19 million people in sub-Saharan Africa. It is a low-income country (LIC) ranked 183 of 189 countries on the Human Development Index [3] with limited natural resources [3]. 2017 GBD estimates across all age groups that injuries are responsible for 7.32% of deaths and 6.48% of disability adjusted life years (DALYs) in Burkina Faso, similar to sub-Saharan Africa rates [4].

In common with the least developed countries, research investigating injuries in Burkina Faso has been sparse [5]. Studies that have been conducted tend to be referral facility based often with small case numbers and limited to a single mechanism of injury pattern [6-10]. Road traffic collisions (RTCs) have been investigated specifically, and the World Health Organisation (WHO) has estimated that in 2016 there were 30.5 per 100,000 road traffic fatalities in Burkina Faso, above the average for Africa (26.6) which is the continent with the highest death rate globally [11]. RTC victim data from the tertiary referral hospital in the capital Ouagadougou identified that 87% of road traffic victims attending for tertiary care emergency department were from two wheel motor vehicles; a quarter of these experienced disability beyond 30 days[12].

Broader injury epidemiology studies from Burkina Faso have primarily studied cause of death data obtained through Verbal Autopsy. In the capital, Ouagadougou, a survey within an urban Health and Demographic Surveillance System (HDSS) identified 4.1% of deaths were due to injury [13]. From the rural Nouna HDSSs Verbal Autopsy data, age and sex standardised mortality for external causes of death (the category containing injuries) was almost twice that of the urban comparator in Ougadougou, with the main cause being transport related. Unfortunately, these Verbal Autopsy based surveys do not capture all mechanisms of injury, and they do not allow assessment of non-fatal injury occurrence [14]. Injury has also been characterised as a disease affecting the young, and some population studies of injury in sub-Saharan Africa have even excluded adults over 70 [15]. However, older people represent an important and growing population in LMICs. How and why older people are injured, and the consequences associated with these injuries require further exploration.

Injuries can have a lasting impact on the victims through physical disability, previously shown beyond 30 days in over a quarter of RTC victims in Ouagadougou,[12] but also psychological morbidity. From High-income country (HIC) settings, depression, anxiety and post-traumatic stress are commonly associated with physical injury [16 17]. This includes older populations with worse quality of life, psychological and social health status seen following hip fractures and osteoporotic vertebral fractures [18 19]. Poor mental health is also a risk factor for non-accidental injuries [20 21]. The impact of mental health following injury within Burkina Faso amongst general older adult population has not been studied, with mental health studies limited to vulnerable populations such as sex workers and children exposed to physical violence in Burkina Faso [22 23].

It has been estimated that if LMIC injury care quality could match that of HIC, then 1/3 of all trauma deaths could be avoided [24], It is thus necessary to improve injury epidemiology data from Burkina Faso to inform preventative measures and treatment services. However, the provision of care alone may not be associated with improved outcomes. Such care needs to be responsive to patients needs beyond providing good clinical outcomes in order to engender trust leading to compliance with treatment and encouragement of future injured persons to attend services [25-27]. However, very few studies on the responsiveness of injury care have been done in LMICs [28].

This analysis primarily aimed to assess the incidence of non-fatal injury and variables associated with this amongst older people in rural Burkina Faso, for which little is currently known. Secondary aims were first to describe the incidence of and variables associated with injury related disability, and second, describe patient reported health system responsiveness following injury.

For peer review only - http://bmjopen.bmj.com/site/about/guidelines.xhtml

Methods

We used the STROBE cross-sectional reporting guidelines [29].

Study setting

The study was set in the Nouna HDSS area, in the Boucle du Mouhoun region, north-western Burkina Faso. The HDSS collects annual birth, death and migration data in a well-enumerated population. The HDSS area consists of the market town Nouna and 59 surrounding villages with a total population of around 107,000 [30]. Residents come from multiple ethnic groups, and the major economic activities are farming and animal husbandry. Life expectancy from birth is 58.0 years for men and 61.5 years for women [31]. There is one tarred road running through the area. There are no formal ambulances with emergency transport usually informal via private or taxi motorbike. In rainy season travel can be very difficult.

Study design

This study is an analysis of the CRSN Heidelberg Aging Study dataset (CHAS). The study methodology has been described in detail elsewhere [32 33] . Briefly, this cross-sectional study consists of a population-representative sample of adults ≥40 years of age. Three thousand older adults were randomly sampled from the 2015 Nouna HDSS census. In all villages (n=6) with fewer than 50 adults aged over 40, all adults were selected to take part. In all other villages, a random sample of households with at least one person over 40 years old was drawn. Then within each selected household, one age-eligible adult was randomly selected to complete the survey, which was administered to them by trained data collectors. Data collection was performed using Open Data Kit (ODK) software on tablet computers at the participants' residence between May and July 2018 [34]. Interviews were conducted either in French or translated into Dioula by the interviewers.

Variables

The household survey contained questions on age, sex, education, marital status, household assets, experience of injury in past 12 months including mechanism and associated disability, reasons for last health facility visit, and questions covering the WHO health system responsiveness domains [25] derived from other surveys used in sub-Saharan Africa [35-37]. Injury data was self-reported and injuries were not independently verified. Anxiety was assessed using the Generalised Anxiety Disorder question (GAD-2) score, [38] and depression using Patient Health Questionnaire (PHQ- 9) [39 40]. Quality of life was measured using the validated EuroHIS 8-item version of WHOQOL[41 42]. Disability was measured using the 12 item WHO Disability Assessment Schedule, version 2 (WHODAS-II) disability score [42 43]. The Fried frailty score was constructed from questions on weight loss in the past year, self-reported activity and levels of exhaustion, combined with measures of walking speed and grip strength [44 45].

Outcome variables

The main outcome variables were whether injured or not in the preceding 12 months, or if injured, whether disabled as a result of the injury. Participants reported whether they had any event where they suffered from bodily injury in the last 12 months. For those reporting yes, the cause of injury was reported along with the question "did you suffer a physical disability as a result of being injured?".

Mechanism of injury

Mechanism of injury was captured as either fall, struck/hit by an object, cut/stabbed, gunshot, fire/heat burn, drowning/near-drowning, poisoning, animal bite, electric shock or other specified by free text. Injury mechanisms with fewer than eight or cases were combined as "other" for analyses. Those who fell reported whether this was at or higher than ground level.

Demographic characteristics

Marital status was categorised as married/cohabiting or single/widowed/divorced. Educational level was categorised as no education or any education. Participants were asked 37 questions on household assets and dwelling characteristics; from these, wealth quintiles were derived from the Filmer and Pritchett first principal component method [46].

Definitions of disease states

Participants were defined as having symptoms of anxiety based on a GAD-2 score ≥3 [38]. Participants scoring ten or more on PHQ-9 were categorised as having depressive symptoms in this analysis [40]. The calculation of the Fried score used in this study has been described previously [32]. For this analysis participants were dichotomised as robust or prefrail/frail. WHODAS-II disability score was

For peer review only - http://bmjopen.bmj.com/site/about/guidelines.xhtml

normalised to a 0-100 scale, where 0 equates to no disability and 100 the worst disability. Quality of life [41 42] was similarly normalised to a 0-100 scale, with 100 denoting the best quality of life.

Health system experience and responsiveness
Regardless of when it occurred, the reason for the most recent episode of health seeking was recorded and classified as either injury or another reason. Participants answering this question were not necessarily the same as those injured in the previous 12 months who may have sought care for another reason subsequent to their injury. There were, therefore, two injury question groups in this study. The first to determine annual injury incidence and characteristics, the second to determine those for whom the last health care visit followed an injury. Appendix Figure 1 illustrates how these overlapping but distinct question groups are reported. Those who had sought care were asked health system responsiveness questions, including: (i) confidence in receiving effective treatment if very sick tomorrow, dichotomised as very/somewhat vs. not very/not at all; (ii) the overall view of the health system, dichotomised as needs to be rebuilt/major changes needed vs. only minor changes needed; (iii) trust in the skills and abilities of the healthcare worker at the facility dichotomised as 1. very much, quite a bit or some and 2. very little or not at all; (iv) ease or difficulty in following provider's advice dichotomised as 1. very easy, easy or fair and 2. hard or very hard; and (v) opinion of care provider's knowledge and skills, experience of being involved in making decisions for treatment, ability of provider to explain things in a way they could understand and how well the received care met health needs were all dichotomised into positive responses 1. excellent, very good, or good and negative responses 2. fair or poor. These variables were dichotomised for ease of interpretation.

Patient and public involvement statement
Participants were not directly involved in planning the study; results of this and other HDSS studies are regularly fed back to participants in the HDSS site.

Statistical analysis
All analyses were done using SPSS v26 (IBM, New York, USA). We first described all variables using mean and standard deviation (SD), or median and interquartile range (IQR), for normally and non-normally distributed continuous variables, and count and proportion (95% CI) for categorical variables.

We used multivariable logistic regression to explore the associations between the main outcome variables and demographic characteristics or disease states. Odds ratios and 95% confidence intervals are presented. All variables were included in the model. Figures were produced using the R package ggplot2 [47].

Associations between seeking care for an injury or another reason and healthcare experience and health system responsiveness were tested using the Mann-Whitney U test for the non-normally distributed continuous variables. Sample sizes are stated for each analysis.

For peer review only

Results:

The median age of respondents was 52 years (IQR 45 – 62), females made up 50.7% (1534/3028) of the population, educational attainment was low, with only 15.6% (472/3028) having any schooling at all (Table 1).

Of those completing the survey, 7.7% (232/3028) reported suffering an injury in the preceding 12 months (Table 1). Of 232 injured in the past 12 months, 105 (45.3%) suffered a disability. In multivariable analyses, younger age, male sex, highest wealth quintile, an abnormal GAD score and lower WHO QOL score were all associated with injury (Table 2).

The most common mechanism of injury was being struck or hit by an object, 32.8% (76/232) (Figure 1). Of those who suffered a fall, 34.6% (9/26) fell from higher than ground level. Exploratory analysis of the association between the mechanism of injury and wealth (Appendix Tables 1 and 2) suggested that the greater odds of being injured in the higher wealth quintile is related to a greater number of falls from a motorcycle or bicycle in this group; 35.8% (19/53) of those falling from a motorcycle or bicycle were from wealth quintile 5 compared to 5.7% (3/53) in quintile 1 (Odds ratio 5.83, 95% CI 1.58 - 21.43).

Falling from a motorcycle or bicycle was the mechanism which most frequently resulted in a disability 27.6% (29/105) (Figure 1). In multivariable analysis (Table 3) only education was significantly negatively associated with odds of disability (Odds Ratio 0.407, 95%CI 0.17–0.997). Compared to being struck or hit by an object, disability was more common amongst those falling (odds ratio 6.4, 95% CI 1.896 – 21.602, p=0.003), falling from a motorbike (odds ratio 3.335, 95% CI 1.429 – 7.78, p=0.005), and other (odds ratio 10.755, 95% CI 3.471 – 33.323, p<0.001).

Across all survey participants, 3.9% (119/3028) of people reported their last reason for seeking care was for an injury. These 119 respondents reported high levels of satisfaction with care following injury (Figure 2). Ninety-six per cent reported being somewhat or very confident of receiving effective treatment if sick tomorrow, 95% reported a good or better opinion of care provider's skills and knowledge and 90% reported that their needs were met well or better. There were no significant differences in these measures between people seeking care for injuries and those seeking care for other reasons (Figure 2 and Appendix Table 3).

Those seeking care following injury reported shorter median wait times (10 minutes vs 20 minutes, P=0.002) before consultation and longer consultation times (20 minutes vs 15 minutes P=0.002) than those seeking care for another reason. Those seeking care for injury were also more likely to be asked to return to the health facility at a later date, 81.4% (95% CI 73.1% – 87.9%) vs. 54.8% (95% CI 49.9% – 53.6%) (Figure 2). There was a non-significant trend for those seeking injury care to be more likely to borrow or sell to pay for the care episode, compared to those seeking care for other reasons, 21.2% (95% CI 14.7% - 29.7%) vs. 14.3% (95% CI 13.0% - 15.6%).

For peer review only

Discussion:

This study demonstrates that injuries are prevalent in this older adult rural LMIC population. In this cross sectional study injuries were more prevalent in those who were male, or of younger age, or wealthier socioeconomic status; the latter is possibly linked to motorcycle ownership. Those with injuries were more likely to suffer from anxiety, or report a worse quality of life. Almost half of those reporting an injury reported disability as a consequence, which was more common in males and those with lower educational attainment, but not associated with frailty. However, in this cross sectional survey, we are unable to demonstrate causality. Patient satisfaction with the health system for treatment of an injury was generally high. Having to sell or borrow to pay for care was more common than for non-traumatic health care visits, although this did not reach significance. There is little empirical data published on injury prevalence and care within Burkina Faso, particularly in older people. This study can aid researchers and policymakers in understanding the burden to address prevention and avenues for further research.

Globally, poorer populations bear increased injury burden, [48] including amongst urban populations [49] and those sustaining unintentional injury [15]. This findings is perhaps due to those of lower SES being exposed to less safe working conditions. Interestingly, we found SES to be positively associated with injury occurrence; potentially, in this rural context, it is likely that relative wealth provided access to motorcycles or bicycles that may have been unaffordable for poorer groups. Further research to prove this hypothesis could have implications for road safety initiatives, particularly if access to motorised transport increases.

Although in an older population, this study found the incidence of injury, 7.7%, was comparable to other sub-Saharan African settings such as in rural Tanzania, rural Rwanda, rural Nigeria, Sudan, Sierra Leone and Kenya where studies have shown prevalence ranges from 4.3 – 15.2% [15 49-53]. Other studies from Sub-Saharan Africa have also found injuries to be more common in younger [15 53] or male [15 49 50 52] members of the population. Indeed, male sex is consistently associated with injury globally, with multiple possible contributing factors including alcohol use, dangerous occupations, or risk taking behaviour [54] – unfortunately, none of these were evaluated in CHAS.

Anxiety and reduced quality of life were associated with the occurrence of injury although no association was seen with depression. Whilst this cross sectional survey could not demonstrate causality, others have shown adverse mental health outcomes to be sequelae of physical injury and include post-traumatic stress disorder, depression and anxiety [16 17 55]. Whilst research exists in high-income settings, further research into the adverse mental health associations with injury in this and other LMIC contexts is warranted. Studies are needed to both establish the scale of burden, whether associations are causal and the direction of the relationship. Development of culturally specific tools for evaluating post physical trauma mental health in African populations is also required [56].

Almost half those injured reported disability (although not defined) as a consequence of their injury, and reporting disability after injury was associated with lower educational status. Other sub-Saharan African studies have reported varying levels of disability after injury, for example, 31.7% in Rwanda [51], and 11% in Sudan [57]. Disability can be more prevalent in rural compared to urban settings [58], amongst the uneducated [57], and in adults over 60 [58]. The different questions employed in these studies makes direct comparison difficult although the high incidence of disability we found may be due to studying an older population with less physical reserve.

In our study, no association between disability and frailty was seen in the population who had been injured. A lower baseline physical function may affect the threshold for self-reported disability. The non-frail population, with low educational attainment, may also have been more dependent on physical labour than other studies and thus more sensitive to limitations to physical function. The association between disability and lower levels of education seen in our study supports this.

The most common injury mechanisms were being struck or hit by an object, falling from a motorcycle or bicycle, being cut or stabbed, or falling. In rural environments, injuries can commonly be a consequence of agricultural activity. This was the leading contributor to injuries in rural Ghana [58]. In Tanzania, cuts or stabs were the most common mechanisms in the rural population studied, and two-thirds of cuts were due to agricultural activity [50]. Transport or road traffic collisions were not a distinct category within our study, given that cars are rarely used in Nouna.

For peer review only - http://bmjopen.bmj.com/site/about/guidelines.xhtml

Interestingly, despite the older age of the CHAS population relative to most previous studies, falls were relatively uncommon, unlike in Kenya [15], Nigeria [52], and Sierra Leone [53] and especially in older persons in Tanzania (aged over 60) [50] Ghana (aged over 60)[58] and Sudan (aged over 45) [49]. The prevalence of frailty in this population is similar to that seen in other sub-Saharan African populations where this has been studied, so the relatively low prevalence of falls (which are associated with frailty) need further investigation [32].

Patients reported a positive experience and high satisfaction with care for both injury and non-injury related consultations. Injured patients experienced shorter wait times and longer consultation time with more frequent requests to return to care than those presenting to healthcare facilities for other reasons. This possibly reflects the urgency of injury care and the need for procedural management such as suturing needing follow-up. Patient satisfaction is influenced by factors such as accessibility, cost, expectation, immediate outcomes and gratitude [28]. However, in LMICs, patient reported satisfaction may not correspond well with other measures of care quality like safe clinical practice or clinical outcomes. For example, high rates of care satisfaction have been reported, across multiple LMICs, with consultations in which most essential clinical actions were not performed [28].

As a time critical condition in an economically poor population there is a risk of injury causing impoverishment. Out-of-pocket expenditure for healthcare is commonplace in Burkina Faso [59]. The economic burden of trauma and injuries in LMICs is high through direct medical costs such as medicines, non-medical costs such as transport and indirect costs such as loss of income [60]. Significant economic benefit could derive from reducing injury burden [61]. In Burkina Faso, CT scan access in Ouagadougou was limited by lack of funding when indicated in 20% of cases [10]. Indeed, perceived and actual costs of care are a well noted factor in delaying access to quality care after injury [26 27 51 57 62]. In CHAS, we found a non-significant trend that patients more commonly needed to borrow or sell following injury than other conditions. This could be compounded by the effect of injury related disability on economic productivity. While CHAS did not capture costs directly, others have found spending on injuries in Nouna HDSS to be higher than other conditions such as malaria and chronic disease [63]. For conditions such as injury, which are unpredictable and high cost, community insurance schemes have been mooted as a way of limiting catastrophic expenditure [64]. Attempts to introduce such schemes in Nouna have suffered high dropout rates, possibly driven by fears around high cost and poor quality of health services [65]. There is evidence from Nouna that individuals enrolled on health insurance schemes received poorer quality healthcare services [66]. Such schemes, limited by low enrolment and selection bias, have failed to make a difference to overall population health including mortality [67 68].

Limitations:
This study has several limitations. The study was cross sectional, which limits the causal interpretation of our findings. Injury severity was not assessed, nor was a clear definition of injury or disability provided. No minimum severity or clear definition can lead to inclusion of trivial injuries, overestimation of burden, and a lack of comparability across studies.

The time of injury was not reported. Recall loss is well established for community injury household surveys, particularly with respect of less severe injuries (causing less than 30 days disability)[50 69 70]. Some studies, therefore, use shorter recall time frames for their survey [52], or extrapolate minor injuries from the last 30 days reported incidence to calculate annual incidence for minor injuries [58]. Observing, confirming or correcting for recall was not possible in this study and we, therefore, may have underestimated the true incidence. As some injuries are known to be seasonal in Burkina Faso, this known recall bias may mean such injuries could have been misrepresented [8].

Intentional injuries and domestic violence were not specifically differentiated from being struck or cut. Similarly, road traffic collision was not a separate category, limited to falling from a motorcycle or bicycle, perhaps justified by the low development status of rural Nouna. As RTCs are the main cause for Urban referral facility trauma care in Burkina Faso [9 10] and the only injury related SDG (SDG 3.6)[71] distinguishing these categories could allow future studies to compare within and across countries to inform preventative lessons and strategies.

This study was limited to a rural population. Urban and rural populations in sub-Saharan Africa experience differing burdens of injury [49 50]. Future urban comparisons could add perspective to inform national preventative and research strategies. This study was also confined to older adults and the injury burned for children and young adults is unknown. Fatal injuries were also not captured.

For peer review only - http://bmjopen.bmj.com/site/about/guidelines.xhtml

To build on these findings future research focussed on injuries could include fatal injuries, across the full population, with rural and urban comparison, capturing the time of injury relative to interview, with specific definitions for injury, disability and mechanism of injury categories, and matched to health system utilisation. This would help further understand the burden in Burkina Faso to inform preventative lessons and strategies as well as plan health system response. Nevertheless, this study adds valuable insight into a relatively under researched topic in a country where little about injury burden or health care experience is known.

Conclusion:
This study has demonstrated the importance of injury burden in this older adult rural LMIC population contributing to the limited available published literature on this subject. Further research could inform preventative strategies including safer farming methods and the role of road traffic collisions, enable better understanding the association between adverse mental health and injury in this population, and strengthen health system readiness to provide quality care.

For peer review only

Table 1 Demographic information and disease states for those injured or not in preceding 12 months, and for those with or without a disability following injury.

Variable and statistical test for comparisons		All %<br><br>Sample size= 3028, apart from age and marital status (both N=3026), WHO DAS (N=3027) and frailty (N=2806)	Not injured in past 12 months (%)<br><br>Sample size=2796, apart from age, marital status (both N=2794), WHO DAS (N=2795) and frailty (N=2607)	Injured in past 12 months (%)<br><br>Sample size=232, apart from frailty (N=199)	Injured in past 12 months – No disability (%)<br><br>Sample size=127, apart from frailty (N=108)	Injured in past 12 months and suffered a disability (%)<br><br>Sample size=105, apart from frailty (N=91)
Median age (years) (IQR)		52 (45 – 62)	52 (45 – 62)	50 (45 – 60)	49 (44 – 61)	51 (45 – 58.5)
Female Sex (95% CI)		50.3 (48.6 - 52.1)	51.5 (49.7 – 53.4)	36.2 (30.3 – 42.6)	40.9 (32.8 – 49.6)	30.5 (22.5 – 39.8)
Marital status (95% CI)	Married/cohabiting	75.7 (74.1 – 77.2)	75.4 (73.8 – 77.0)	78.4 (72.7 – 83.3)	73.2 (64.9 – 80.2)	84.8 (76.7 – 90.4)
	Other	24.3 (22.8 – 25.9)	24.5 (23.0 – 26.2)	21.6 (16.8 – 27.3)	25.2 (19.8 – 35.1)	15.2 (9.6 – 23.3)
Socio-Economic Status	Quintile 1	19.9	20.2	16.4	17.3	15.2
	Quintile 2	19.8	20.1	15.9	19.7	11.4
	Quintile 3	20.0	19.8	22.4	19.7	25.7
	Quintile 4	20.3	20.2	20.7	17.3	24.8
	Quintile 5	20.0	19.6	24.6	26.0	22.9
What is the highest level of education you have completed? (95% CI)	No Formal Schooling	84.4 (83.1 – 85.7)	84.9 (83.5 – 86.2)	78.4 (72.7 – 83.3)	72.4 (64.1 – 79.5)	85.7 (77.8 – 91.2)
	Any schooling	15.6 (14.3 – 16.9)	15.1 (13.8 – 16.5)	21.6 (16.8 – 27.3)	27.6 (20.5 – 35.9)	14.3 (8.9 – 22.2)
Patient Health Questionnaire (PHQ- 9) depression score (95% CI)	Normal or mild	91.9 (90.9 – 92.9)	92.0 (91.0 – 93.0)	91.0 (86.6 – 94.0)	90.6 (84.2 – 94.5)	90.5 (84.5 – 95.4)
	Moderate – severe*	8.1 (7.1 – 9.1)	8.0 (7.0 – 9.0)	9.0 (6.0 – 13.4)	9.4 (5.5 – 15.8)	8.5 (4.6 – 15.5)
Normalised WHO Disability Assessment Schedule 2.0 (median score IQR)		8.3 (2.1 – 22.9)	8.3 (2.1 – 22.9)	8.3 (2.1 – 25)	8.3 (2.1 – 22.9)	10.4 (2.1 – 27.1)
Normalised WHO Quality of Life (median and IQR)		59.4 (46.9 – 65.6)	59.4 (46.9 – 59.4)	56.3 (43.8 – 56.3)	56.3 (46.9 – 62.5)	56.3 (40.6 – 65.6)
Generalised Anxiety Disorder score (95% CI)	Abnormal	11.6 (10.5 – 12.8)	10.6 (9.5 – 11.8)	23.3 (18.3 – 29.1)	22.0 (15.7 – 30.0)	24.8 (17.5 – 33.8)
	Normal	88.4 (87.3 – 89.5)	89.4 (88.2 – 90.5)	76.7 (70.9 – 81.7)	78.0 (70.7 – 84.3)	75.2 (66.2 – 82.5)
Fried frailty score (95% CI)	Robust	45.3 (43.4 – 47.1)	45.3 (43.4 – 47.2)	44.7 (38.0 – 51.7)	38.0 (29.4 – 47.4)	52.7 (42.6 – 62.7)
	Pre-frail / Frail	54.7 (52.9 – 56.6)	54.7 (52.8 – 56.6)	55.3 (48.3 – 62.0)	62.0 (52.6 – 70.6)	47.3 (37.3 – 57.4)
*Score of 10 or more						

For peer review only - http://bmjopen.bmj.com/site/about/guidelines.xhtml

Table 2 - Logistic regression model for dependent variable of injury in last 12 months

		Odds Ratio	95% CI	P
Age		0.973	0.956 - 0.991	0.003
Sex	Male (ref)			
	Female	0.436	0.310 - 0.613	<0.001
Marital status	Married or cohabiting (ref)			
	Not married or cohabiting	1.247	0.812 - 1.917	0.313
Wealth Quintile	Quintile 1 (ref)			
	Quintile 2	1.011	0.597 - 1.712	0.967
	Quintile 3	1.453	0.886 - 2.382	0.138
	Quintile 4	1.367	0.823 - 2.272	0.227
	Quintile 5	1.795	1.079 - 2.985	0.024
Educational level	No Formal Schooling (ref)			
	Any Schooling	1.107	0.749 - 1.636	0.609
Patient Health Questionnaire (PHQ- 9) depression score	Normal or Mild (ref)			
	Moderate or Severe	0.559	0.295 - 1.059	0.075
Generalised Anxiety Disorder score	Normal (ref)			
	abnormal	2.921	1.963 - 4.347	<0.001
Normalised WHO Quality of Life score		0.981	0.97 - 0.993	0.002
Normalised WHO Disability Assessment Schedule 2.0 (0-100)		1.004	0.992 - 1.016	0.512
Frailty	Robust (ref)			
	Pre-frail / Frail	0.967	0.704 - 1.327	0.834
**N = 2803**				

For peer review only

Table 3 - Multivariable logistic regression model for dependent variable of disability following injury in last 12 months including mechanism of injury

		Odds Ratio	95% CI	P
Age		1.016	0.977 – 1.057	0.426
Sex	Male (ref)			
	Female	0.471	0.215 – 1.033	0.06
Marital status	Married or cohabiting (ref)			
	Not married or cohabiting	0.433	0.15 – 1.25	0.122
Wealth Quintile	Quintile 1 (ref)			
	Quintile 2	0.636	0.187 – 2.167	0.469
	Quintile 3	1.09	0.368 – 3.226	0.876
	Quintile 4	2.05	0.666 – 6.306	0.211
	Quintile 5	1.521	0.503 – 4.596	0.457
Educational level	No Formal Schooling (ref)			
	Any Schooling	0.407	0.166 – 0.997	0.049
Patient Health Questionnaire (PHQ- 9) depression score	Normal or Mild (ref)			
	Moderate or Severe	0.426	0.107 – 1.689	0.225
Generalised Anxiety Disorder 2.0 score (0-100)	Normal (ref)			
	Abnormal	0.823	0.36 – 1.882	0.644
Normalised WHO Quality of Life score		1.012	0.985 – 1.039	0.387
Normalised WHO DAS 2.0 score (0-100)		1.023	0.997 – 1.05	0.084
Frailty	Robust (ref)			
	Pre-frail / Frail	0.562	0.284 – 1.112	0.098
Mechanism of injury –	Struck or hit by object (ref)			
	Fall	6.4	1.896 – 21.602	0.003
	Fall from motorbike	3.335	1.429 – 7.78	0.005
	Cut or stabbed	2.426	0.949 – 6.201	0.064
	Other	10.755	3.471 – 33.323	<0.001
**N = 199**				

For peer review only - http://bmjopen.bmj.com/site/about/guidelines.xhtml

Figure legends

Figure 1 – Mechanism of injury according to sex and associated disability
Figure 2 - Opinion and experience of healthcare received by those seeking care following an injury and those seeking care for another reason at last visit.

Appendix Figure 1 - The survey questions determining primary and secondary outcomes.

For peer review only

References:

1. Haagsma JA, Graetz N, Bolliger I, et al. The global burden of injury: incidence, mortality, disability-adjusted life years and time trends from the Global Burden of Disease study 2013. *Injury prevention : journal of the International Society for Child and Adolescent Injury Prevention* 2016;22(1):3-18. doi: 10.1136/injuryprev-2015-041616 [published Online First: 2015/12/05]
2. Gosselin RA, Spiegel DA, Coughlin R, et al. Injuries: the neglected burden in developing countries. *Bull World Health Organ* 2009;87(4):246-46a. [published Online First: 2009/06/25]
3. Bank. W. The world bank in Burkina Faso 2019 [Available from: http://www.worldbank.org/en/country/burkinafaso/overview
4. Global Health Data Exchange: Global Burden of Disease Results Tool [Available from: http://ghdx.healthdata.org/gbd-results-tool accessed 06/08/2020.
5. Mc Sween-Cadieux E, Dagenais C, Ridde V. A deliberative dialogue as a knowledge translation strategy on road traffic injuries in Burkina Faso: a mixed-method evaluation. *Health Res Policy Syst* 2018;16(1):113. doi: 10.1186/s12961-018-0388-8 [published Online First: 2018/11/22]
6. Ouedraogo R, Konsem T, Gyebre Y, et al. Experience with head and neck missile injuries at the yalgado university teaching hospital, ouagadougou, burkina faso. *J West Afr Coll Surg* 2012;2(4):1-9. [published Online First: 2012/10/01]
7. Sawadogo N, Garanet F, Traoré D, et al. [Morbidity of adolescents at the Kaya Regional Hospital Center (Burkina Faso)]. *Mali Med* 2018;33(1):29-34. [published Online First: 2018/11/30]
8. Dakouré PW, Diallo M, Traoré AC, et al. Trauma related to falls from trees treated in a specialized trauma centre in Burkina-Faso-one hundred and six cases treated in one year. *Int Orthop* 2015;39(12):2451-6. doi: 10.1007/s00264-015-2966-5 [published Online First: 2015/08/25]
9. Konsem T, Millogo M, Ouedraogo YI, et al. [Epidemiology of maxillo-facial traumatism s sequels at stomatology and maxillo-facial surgery service of Yalgado Ouedraogo University Hospital Center]. *Odontostomatol Trop* 2016;39(156):66-72. [published Online First: 2016/12/01]
10. Kabore AF, Ouedraogo A, Ki KB, et al. Head Computed Tomography Scan in Isolated Traumatic Brain Injury in a Low-Income Country. *World Neurosurg* 2017;107:382-88. doi: 10.1016/j.wneu.2017.07.160 [published Online First: 2017/08/23]
11. World Health Organization. Global status report on road safety 2018., 2018.
12. Bonnet E FA, Nikiema A, Ouedraogo M, Lechat L, Ridde V. . Se protéger en circulation réduit la gravité des blessures., 2016.
13. Soura AB, Lankoande B, Millogo R, et al. Comparing causes of death between formal and informal neighborhoods in urban Africa: evidence from Ouagadougou Health and Demographic Surveillance System. *Global health action* 2014;7:25523. doi: 10.3402/gha.v7.25523 [published Online First: 2014/11/08]
14. Streatfield PK, Khan WA, Bhuiya A, et al. Mortality from external causes in Africa and Asia: evidence from INDEPTH Health and Demographic

Surveillance System Sites. *Global health action* 2014;7:25366. doi: 10.3402/gha.v7.25366 [published Online First: 2014/11/08]
15. Gathecha GK, Ngaruiya C, Mwai W, et al. Prevalence and predictors of injuries in Kenya: findings from the national STEPs survey. *BMC public health* 2018;18(Suppl 3):1222-22. doi: 10.1186/s12889-018-6061-x
16. Wiseman T, Foster K, Curtis K. Mental health following traumatic physical injury: An integrative literature review. *Injury* 2013;44(11):1383-90. doi: 10.1016/j.injury.2012.02.015
17. Zatzick D, Roy-Byrne P, Russo J, et al. A randomized effectiveness trial of stepped collaborative care for acutely injured trauma survivors. *Arch Gen Psychiatry* 2004;61(5):498-506. doi: 10.1001/archpsyc.61.5.498 [published Online First: 2004/05/05]
18. Al-Sari UA, Tobias J, Clark E. Health-related quality of life in older people with osteoporotic vertebral fractures: a systematic review and meta-analysis. *Osteoporos Int* 2016;27(10):2891-900. doi: 10.1007/s00198-016-3648-x [published Online First: 2016/06/06]
19. Peeters CM, Visser E, Van de Ree CL, et al. Quality of life after hip fracture in the elderly: A systematic literature review. *Injury* 2016;47(7):1369-82. doi: 10.1016/j.injury.2016.04.018 [published Online First: 2016/05/15]
20. Schecter WP, Klassen C, O'Connor P, et al. Suicide: the unmet challenge of the trauma system. *Archives of surgery (Chicago, Ill : 1960)* 2005;140(9):902-4. doi: 10.1001/archsurg.140.9.902 [published Online First: 2005/09/24]
21. Zatzick DF, Kang SM, Kim SY, et al. Patients with recognized psychiatric disorders in trauma surgery: incidence, inpatient length of stay, and cost. *J Trauma* 2000;49(3):487-95. doi: 10.1097/00005373-200009000-00017 [published Online First: 2000/09/26]
22. Cange CW, Wirtz AL, Ky-Zerbo O, et al. Effects of traumatic events on sex workers' mental health and suicide intentions in Burkina Faso: a trauma-informed approach. *Sex Health* 2019;16(4):348-57. doi: 10.1071/sh17213 [published Online First: 2019/07/12]
23. Ismayilova L, Gaveras E, Blum A, et al. Maltreatment and Mental Health Outcomes among Ultra-Poor Children in Burkina Faso: A Latent Class Analysis. *PloS one* 2016;11(10):e0164790. doi: 10.1371/journal.pone.0164790 [published Online First: 2016/10/21]
24. Mock C, Joshipura M, Arreola-Risa C, et al. An estimate of the number of lives that could be saved through improvements in trauma care globally. *World J Surg* 2012;36(5):959-63. doi: 10.1007/s00268-012-1459-6 [published Online First: 2012/03/16]
25. Darby C VN, Murray CJ, De Silva A. World Health Organization (WHO): Strategy on measuring responsiveness, 2003.
26. Whitaker J, Nepogodiev D, Leather A, et al. Assessing barriers to quality trauma care in low and middle-income countries: A Delphi study. *Injury* 2019 doi: 10.1016/j.injury.2019.12.035 [published Online First: 2019/12/31]
27. Odland ML, Whitaker J, Nepogodiev D, et al. Identifying, Prioritizing and Visually Mapping Barriers to Injury Care in Rwanda: A Multi-disciplinary Stakeholder Exercise. *World Journal of Surgery* 2020 doi: 10.1007/s00268-020-05571-6

For peer review only

For peer review only - http://bmjopen.bmj.com/site/about/guidelines.xhtml

28. Kruk ME, Gage AD, Arsenault C, et al. High-quality health systems in the Sustainable Development Goals era: time for a revolution. *Lancet Glob Health* 2018 doi: 10.1016/s2214-109x(18)30386-3 [published Online First: 2018/09/10]
29. von Elm E, Altman DG, Egger M, et al. The Strengthening the Reporting of Observational Studies in Epidemiology (STROBE) statement: guidelines for reporting observational studies. *J Clin Epidemiol* 2008;61(4):344-9. doi: 10.1016/j.jclinepi.2007.11.008 [published Online First: 2008/03/04]
30. Sie A, Louis VR, Gbangou A, et al. The Health and Demographic Surveillance System (HDSS) in Nouna, Burkina Faso, 1993-2007. *Glob Health Action* 2010;3 doi: 10.3402/gha.v3i0.5284 [published Online First: 2010/09/18]
31. Odhiambo Sewe M, Bunker A, Ingole V, et al. Estimated Effect of Temperature on Years of Life Lost: A Retrospective Time-Series Study of Low-, Middle-, and High-Income Regions. *Environ Health Perspect* 2018;126(1):017004-04. doi: 10.1289/EHP1745
32. Witham MD, Davies JI, Bärnighausen T, et al. Frailty and physical performance in the context of extreme poverty: a population-based study of older adults in rural Burkina Faso. *Wellcome Open Res* 2019;4:135-35. doi: 10.12688/wellcomeopenres.15455.1
33. Odland ML, Payne C, Witham MD, et al. Epidemiology of multimorbidity in conditions of extreme poverty: a population-based study of older adults in rural Burkina Faso. *BMJ global health* 2020;5(3):e002096-e96. doi: 10.1136/bmjgh-2019-002096
34. Hartung C. Open Data Kit: Tools to Build Information Services for Developing Regions. 2010
35. Ratcliffe HL, Bell G, Awoonor-Williams K, et al. Towards patient-centred care in Ghana: health system responsiveness, self-rated health and experiential quality in a nationally representative survey. *BMJ Open Qual* 2020;9(2):e000886. doi: 10.1136/bmjoq-2019-000886
36. Baltussen RM, Yé Y, Haddad S, et al. Perceived quality of care of primary health care services in Burkina Faso. *Health Policy Plan* 2002;17(1):42-8. doi: 10.1093/heapol/17.1.42 [published Online First: 2002/02/28]
37. Miller JS, Mhalu A, Chalamilla G, et al. Patient satisfaction with HIV/AIDS care at private clinics in Dar es Salaam, Tanzania. *AIDS care* 2014;26(9):1150-4. doi: 10.1080/09540121.2014.882487 [published Online First: 2014/02/07]
38. Plummer F, Manea L, Trepel D, et al. Screening for anxiety disorders with the GAD-7 and GAD-2: a systematic review and diagnostic metaanalysis. *Gen Hosp Psychiatry* 2016;39:24-31. doi: 10.1016/j.genhosppsych.2015.11.005 [published Online First: 2016/01/01]
39. Spitzer RL, Kroenke K, Williams JB. Validation and utility of a self-report version of PRIME-MD: the PHQ primary care study. Primary Care Evaluation of Mental Disorders. Patient Health Questionnaire. *JAMA* 1999;282(18):1737-44. doi: 10.1001/jama.282.18.1737 [published Online First: 1999/11/24]
40. Kroenke K, Spitzer RL, Williams JB. The PHQ-9: validity of a brief depression severity measure. *J Gen Intern Med* 2001;16(9):606-13. doi:

For peer review only - http://bmjopen.bmj.com/site/about/guidelines.xhtml

10.1046/j.1525-1497.2001.016009606.x [published Online First: 2001/09/15]
41. Schmidt S, Muhlan H, Power M. The EUROHIS-QOL 8-item index: psychometric results of a cross-cultural field study. *Eur J Public Health* 2006;16(4):420-8. doi: 10.1093/eurpub/cki155 [published Online First: 2005/09/06]
42. Gomez-Olive FX, Schroders J, Aboderin I, et al. Variations in disability and quality of life with age and sex between eight lower income and middle-income countries: data from the INDEPTH WHO-SAGE collaboration. *BMJ Glob Health* 2017;2(4):e000508. doi: 10.1136/bmjgh-2017-000508 [published Online First: 2018/01/16]
43. Garin O, Ayuso-Mateos JL, Almansa J, et al. Validation of the "World Health Organization Disability Assessment Schedule, WHODAS-2" in patients with chronic diseases. *Health Qual Life Outcomes* 2010;8:51. doi: 10.1186/1477-7525-8-51 [published Online First: 2010/05/21]
44. Fried LP, Tangen CM, Walston J, et al. Frailty in older adults: evidence for a phenotype. *J Gerontol A Biol Sci Med Sci* 2001;56(3):M146-56. doi: 10.1093/gerona/56.3.m146 [published Online First: 2001/03/17]
45. Witham MDD, J.I. Barning, T. Boun, M. Manne-Goi. Frailty and physical performance in the context of extreme poverty: a population-based study of older adults in rural Burkina Faso. *Wellcome Open Res* 2019
46. Filmer D, Pritchett LH. Estimating wealth effects without expenditure data--or tears: an application to educational enrollments in states of India. *Demography* 2001;38(1):115-32. doi: 10.1353/dem.2001.0003 [published Online First: 2001/03/03]
47. Wickham H. ggplot2: Elegant Graphics for Data Analysis: Springer-Verlag New York 2009.
48. Nantulya VM, Reich MR. Equity dimensions of road traffic injuries in low- and middle-income countries. *Inj Control Saf Promot* 2003;10(1-2):13-20. doi: 10.1076/icsp.10.1.13.14116 [published Online First: 2003/05/30]
49. Tayeb SE, Abdalla S, Mørkve O, et al. Injuries in Khartoum state, the Sudan: a household survey of incidence and risk factors. *International Journal of Injury Control and Safety Promotion* 2014;21(2):144-53. doi: 10.1080/17457300.2013.792283
50. Moshiro C, Heuch I, Åstrøm AN, et al. Injury morbidity in an urban and a rural area in Tanzania: an epidemiological survey. *BMC Public Health* 2005;5(1):11. doi: 10.1186/1471-2458-5-11
51. Petroze RT, Joharifard S, Groen RS, et al. Injury, disability and access to care in Rwanda: results of a nationwide cross-sectional population study. *World J Surg* 2015;39(1):62-9. doi: 10.1007/s00268-014-2544-9 [published Online First: 2014/05/29]
52. Olawale OA, Owoaje ET. Incidence and pattern of injuries among residents of a rural area in South-Western Nigeria: a community-based study. *BMC Public Health* 2007;7(1):246. doi: 10.1186/1471-2458-7-246
53. Stewart K-AA, Groen RS, Kamara TB, et al. Traumatic injuries in developing countries: report from a nationwide cross-sectional survey of Sierra Leone. *JAMA surgery* 2013;148(5):463-69. doi: 10.1001/jamasurg.2013.1341

For peer review only

54. Sorenson SB. Gender disparities in injury mortality: consistent, persistent, and larger than you'd think. *Am J Public Health* 2011;101 Suppl 1(Suppl 1):S353-S58. doi: 10.2105/AJPH.2010.300029 [published Online First: 2011/07/21]
55. World Health Organisation. Injuries and Violence: The Facts., 2014.
56. Wyatt GE, Thames A, Simbayi L, et al. Trauma and mental health in South Africa: Overview. *Psychol Trauma* 2017;9(3):249-51. doi: 10.1037/tra0000144
57. El Tayeb S, Abdalla S, Heuch I, et al. Socioeconomic and disability consequences of injuries in the Sudan: a community-based survey in Khartoum State. *Injury prevention : journal of the International Society for Child and Adolescent Injury Prevention* 2015;21(e1):e56-62. doi: 10.1136/injuryprev-2013-040818 [published Online First: 2013/11/15]
58. Mock CN, Abantanga F, Cummings P, et al. Incidence and outcome of injury in Ghana: a community-based survey. *Bull World Health Organ* 1999;77(12):955-64. [published Online First: 2000/02/19]
59. Beogo I, Huang N, Gagnon MP, et al. Out-of-pocket expenditure and its determinants in the context of private healthcare sector expansion in sub-Saharan Africa urban cities: evidence from household survey in Ouagadougou, Burkina Faso. *BMC research notes* 2016;9:34. doi: 10.1186/s13104-016-1846-4 [published Online First: 2016/01/23]
60. Wesson HKH, Boikhutso N, Bachani AM, et al. The cost of injury and trauma care in low- and middle-income countries: a review of economic evidence. *Health policy and planning* 2014;29(6):795-808. doi: 10.1093/heapol/czt064 [published Online First: 2013/10/05]
61. Kotagal M, Agarwal-Harding KJ, Mock C, et al. Health and economic benefits of improved injury prevention and trauma care worldwide. *PloS one* 2014;9(3):e91862. doi: 10.1371/journal.pone.0091862 [published Online First: 2014/03/15]
62. Wu Y, Zhang W, Zhang L, et al. Non-fatal injuries treated outside a hospital in Hunan, China: results from a household interview survey. *European journal of public health* 2016;27(2):331-34. doi: 10.1093/eurpub/ckw114
63. Su TT, Pokhrel S, Gbangou A, et al. Determinants of household health expenditure on western institutional health care. *Eur J Health Econ* 2006;7(3):199-207. doi: 10.1007/s10198-006-0354-1 [published Online First: 2006/05/05]
64. Meara JG, Leather AJ, Hagander L, et al. Global Surgery 2030: evidence and solutions for achieving health, welfare, and economic development. *Lancet (London, England)* 2015;386(9993):569-624. doi: 10.1016/s0140-6736(15)60160-x [published Online First: 2015/05/01]
65. Dong H, De Allegri M, Gnawali D, et al. Drop-out analysis of community-based health insurance membership at Nouna, Burkina Faso. *Health Policy* 2009;92(2-3):174-9. doi: 10.1016/j.healthpol.2009.03.013 [published Online First: 2009/04/28]
66. Robyn PJ, Fink G, Sié A, et al. Health insurance and health-seeking behavior: evidence from a randomized community-based insurance rollout in rural Burkina Faso. *Soc Sci Med* 2012;75(4):595-603. doi: 10.1016/j.socscimed.2011.12.018 [published Online First: 2012/02/11]

For peer review only - http://bmjopen.bmj.com/site/about/guidelines.xhtml

67. Hounton S, Byass P, Kouyate B. Assessing effectiveness of a community based health insurance in rural Burkina Faso. *BMC Health Serv Res* 2012;12:363. doi: 10.1186/1472-6963-12-363 [published Online First: 2012/10/23]
68. Fink G, Robyn PJ, Sié A, et al. Does health insurance improve health?: Evidence from a randomized community-based insurance rollout in rural Burkina Faso. *J Health Econ* 2013;32(6):1043-56. doi: 10.1016/j.jhealeco.2013.08.003 [published Online First: 2013/10/10]
69. Mock C, Acheampong F, Adjei S, et al. The effect of recall on estimation of incidence rates for injury in Ghana. *International journal of epidemiology* 1999;28(4):750-5. [published Online First: 1999/09/10]
70. Moshiro C, Heuch I, Astrøm AN, et al. Effect of recall on estimation of non-fatal injury rates: a community based study in Tanzania. *Injury prevention : journal of the International Society for Child and Adolescent Injury Prevention* 2005;11(1):48-52. doi: 10.1136/ip.2004.005645
71. World Health Organization. Sustainable Development Goal 3: Health 2015 [Available from: https://www.who.int/topics/sustainable-development-goals/targets/en/.

For peer review only

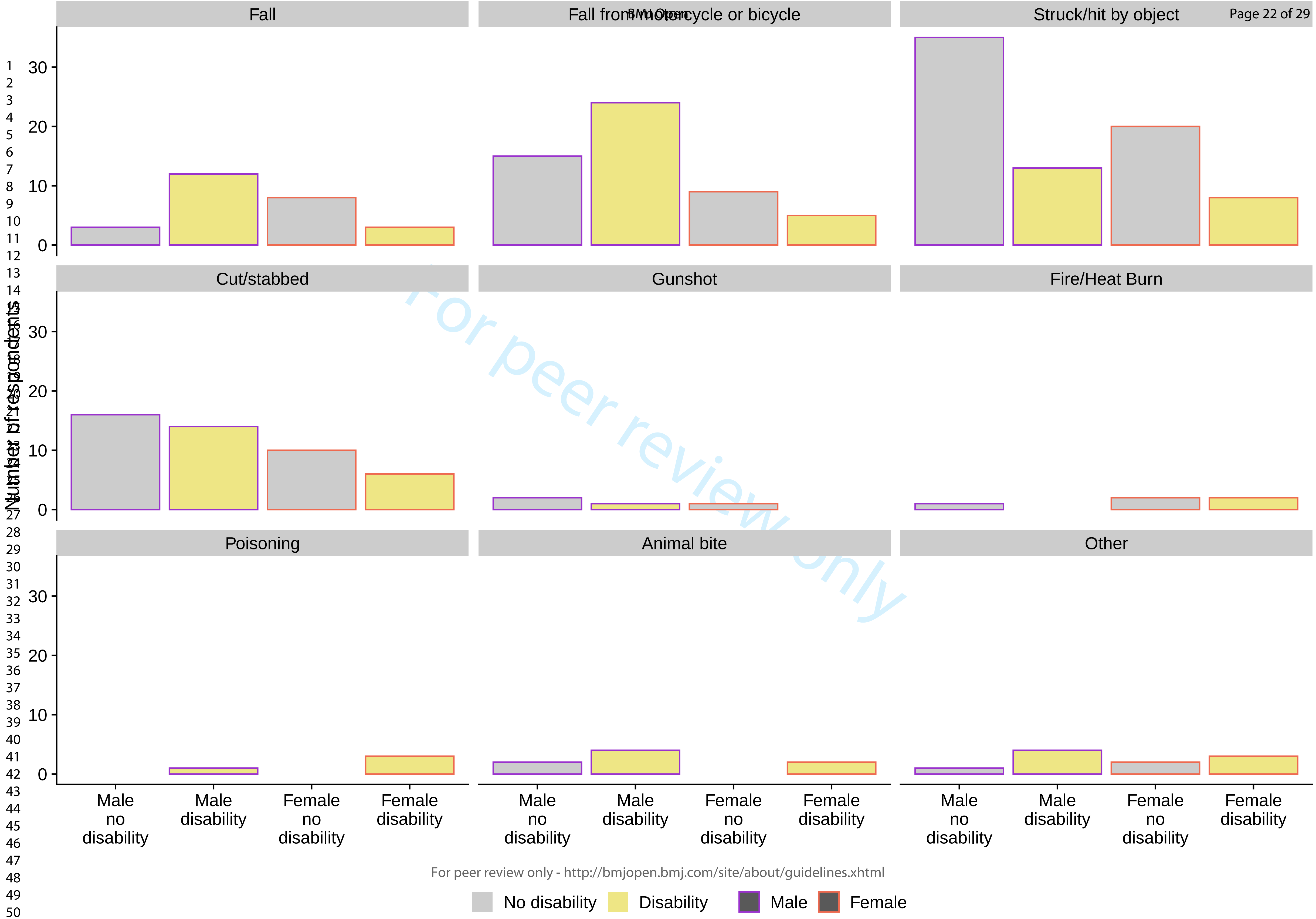

For peer review only - http://bmjopen.bmj.com/site/about/guidelines.xhtml

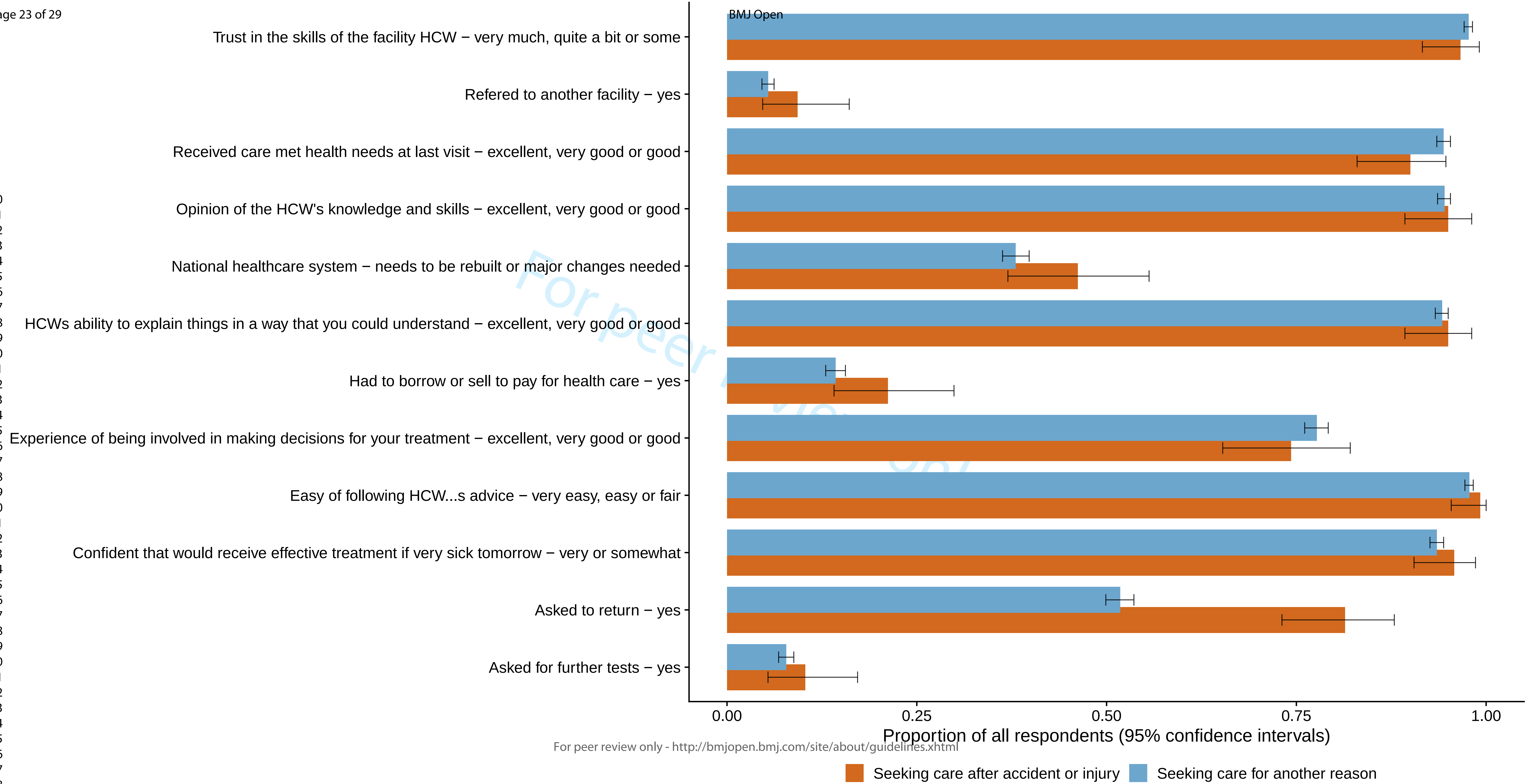

For peer review only - http://bmjopen.bmj.com/site/about/guidelines.xhtml

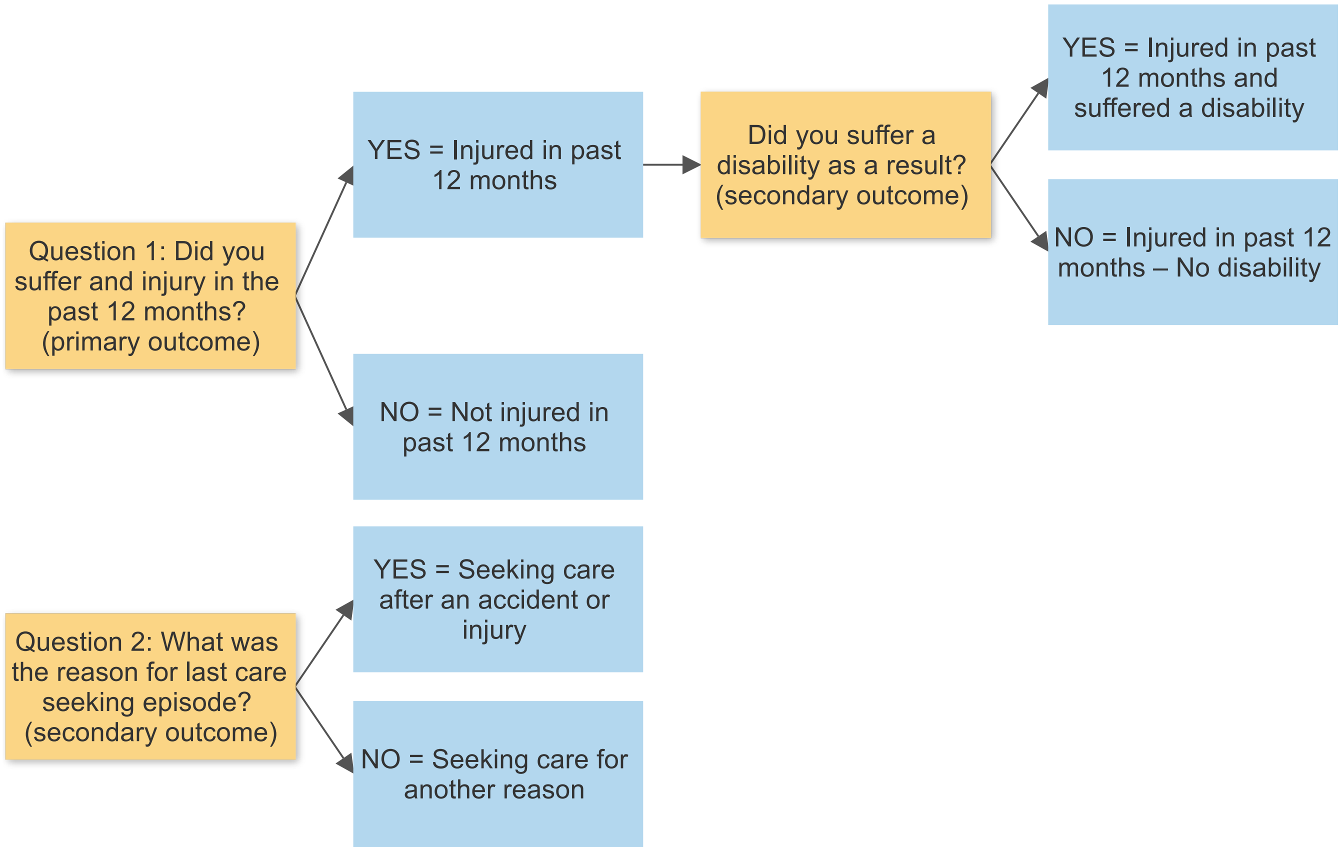
Question 1: Did you suffer and injury in the past 12 months? (primary outcome)
YES = Injured in past 12 months
NO = Not injured in past 12 months
Did you suffer a disability as a result? (secondary outcome)
YES = Injured in past 12 months and suffered a disability
NO = Injured in past 12 months – No disability
Question 2: What was the reason for last care seeking episode? (secondary outcome)
YES = Seeking care after an accident or injury
NO = Seeking care for another reason

Appendices

Appendix table 1 - Mechanism of injury according to wealth quintile, for all those injured in past 12 months with number of males in brackets.

Wealth Quintile	Fall N (n of males)	Fall from motorcycle or bicycle N (n of males)	Struck / hit by object N (n of males)	Cut / stabbed N (n of males)	Gunshot N (n of males)	Fire / Heat Burn N (n of males)	Poisoning N (n of males)	Animal bite N (n of males)	Other N (n of males)
1	6 (4)	3 (3)	10 (7)	11 (5)	1 (1)	1 (0)	0 (0)	2 (1)	4 (2)
2	5 (1)	6 (5)	17 (13)	2 (1)	2 (2)	1 (1)	3 (0)	1 (1)	0 (0)
3	9 (5)	12 (6)	12 (8)	15 (11)	0 (0)	0 (0)	1 (1)	0 (0)	3 (2)
4	3 (3)	13 (10)	20 (11)	5 (5)	0 (0)	2 (0)	0 (0)	2 (2)	3 (1)
5	3 (2)	19 (15)	17 (9)	13 (8)	1 (0)	1 (0)	0 (0)	3 (2)	0 (0)
Total	26 (15)	53 (39)	76 (48)	46 (30)	4 (3)	5 (1)	4 (1)	8 (6)	10 (5)

Appendix table 2 - Binary logistic regression for falling from a motorcycle or bicycle and wealth quintile.

	Odds Ratio	95% C.I.	P Value
Quintile 1 (ref)			
Quintile 2	2.26	0.52 - 9.80	0.277
Quintile 3	3.50	0.91 - 13.42	0.068
Quintile 4	4.33	1.14 - 16.55	0.032
Quintile 5	5.83	1.59 - 21.43	0.008

Appendix table 3 - Opinion and experience of healthcare received by those seeking care following an injury and those seeking care for another reason at last visit.

	Seeking care after an accident or injury % (95% CI) Number responders (N) = 119 unless otherwise stated	Seeking care for another reason n % (95% CI) Number responders (N) = 2882 unless otherwise stated
**Opinion of care received**		
Confident that would receive effective treatment if very sick tomorrow		
Very or somewhat confident	95.8 (90.5 – 98.2)	93.5 (92.6 – 94.4)
Not very or not at all confident	4.2 (1.8 – 9.5)	6.5 (5.6 – 7.4)
Overall view of national healthcare system		
Needs to be rebuilt or major changes needed	46.2 (37.5 – 55.2)	38.0 (36.3 – 39.8) (N=2909)
Only minor changes needed	53.8 (44.9 – 62.5)	62.0 (60.2 – 63.7) (N=2909)
Overall how well did received care meet health needs at last visit?		
Excellent, very good or good	90.0 (83.2 – 94.1)	94.4 (93.5 – 95.2)
Fair or poor	10.0 (5.9 – 16.8)	5.5 (4.8 – 6.5)
Do you trust in the skills and abilities of the HCW at the facility?		
Very much, quite a bit or some.	96.6 (91.7 – 98.7)	97.7 (97.1 – 98.2) (N=2879)
Very little or not at all	3.4 (1.3 – 8.3)	2.3 (1.8 – 2.9) (N=2879)
What is your opinion of the care provider's knowledge and skills?		
Excellent, very good or good	95.0 (89.4 – 97.7)	94.5 (93.3 – 95.0) (N=2873)
Fair or poor	5.0 (2.3 – 10.6)	5.5 (5.0 – 6.7) (N=2873)
What do you think about the provider's ability to explain things in a way that you could understand?		

For peer review only

	Excellent, very good or good	95.0 (89.4 – 97.7)	94.2 (93.3 – 95.0) (N=2864)
	Fair or poor	5.0 (2.3 – 10.6)	5.8 (5.0 – 6.7) (N=2864)
How easy or difficult was it for you to follow the provider's advice?			
	Very Easy, easy or fair	99.2 (95.4 – 99.9)	97.8 (97.3 – 98.3) (N=2877)
	Hard or very hard	0.8 (0.2 – 4.6)	2.2 (1.7 – 2.8) (N=2877)
What do you think about your experience of being involved in making decisions for your treatment?			
	Excellent, very good or good	74.3 (65.6 – 81.5) (N=113)	77.7 (76.1 – 79.2) (N=2748)
	Fair or poor	25.7 (18.5 – 34.4) (N=113)	22.3 (20.8 – 23.9) (N=2748)
**Experience of care processes**			
Did care provider refer to another facility? - Yes		9.3 (5.2 – 15.8)	5.4 (4.6 – 6.3) (N=2872)
Did care provider ask for further tests? - Yes		10.3 (6.0 – 17.1) (N=117)	7.8 (6.9 – 8.8) (N=2859)
Did the care provider ask you to come back? - Yes		81.4 (72.7 – 86.8)	51.8 (50.0 – 53.6) (N=2881)
Had to borrow or sell to pay for health care episode? - Yes		21.2 (14.7 – 29.7) (N=113)	14.3 (13.0 – 15.6) (N=2881)
How long did you wait before your consultation (minutes)? (Median and IQR) (Mann-Witney U)		10 (5-15) (N=118)	20 (10-30) (N=2885)<br>P = 0.002
How much time did you spend with the care provider (minutes)? (Median and IQR) (Mann-Witney U)		20 (15-30) (N=118)	15 (20-25) (N=2885)<br>P = 0.002

For peer review only - http://bmjopen.bmj.com/site/about/guidelines.xhtml

Reporting checklist for cross sectional study.

Based on the STROBE cross sectional guidelines.

		Reporting Item	Page Number
**Title and abstract**			
Title	#1a	Indicate the study's design with a commonly used term in the title or the abstract	1
Abstract	#1b	Provide in the abstract an informative and balanced summary of what was done and what was found	2
**Introduction**			
Background / rationale	#2	Explain the scientific background and rationale for the investigation being reported	4
Objectives	#3	State specific objectives, including any prespecified hypotheses	4
**Methods**			
Study design	#4	Present key elements of study design early in the paper	5
Setting	#5	Describe the setting, locations, and relevant dates, including periods of recruitment, exposure, follow-up, and data collection	5
Eligibility criteria	#6a	Give the eligibility criteria, and the sources and methods of selection of participants.	5
	#7	Clearly define all outcomes, exposures, predictors, potential confounders, and effect modifiers. Give diagnostic criteria, if applicable	5-6
Data sources / measurement	#8	For each variable of interest give sources of data and details of methods of assessment (measurement). Describe comparability of assessment methods if there is more than one	5

For peer review only

		group. Give information separately for for exposed and unexposed groups if applicable.	
Bias	#9	Describe any efforts to address potential sources of bias	5
Study size	#10	Explain how the study size was arrived at	5
Quantitative variables	#11	Explain how quantitative variables were handled in the analyses. If applicable, describe which groupings were chosen, and why	5-6
Statistical methods	#12a	Describe all statistical methods, including those used to control for confounding	6
Statistical methods	#12b	Describe any methods used to examine subgroups and interactions	6
Statistical methods	#12c	Explain how missing data were addressed	6
Statistical methods	#12d	If applicable, describe analytical methods taking account of sampling strategy	NA
Statistical methods	#12e	Describe any sensitivity analyses	6
**Results**			
Participants	#13a	Report numbers of individuals at each stage of study—eg numbers potentially eligible, examined for eligibility, confirmed eligible, included in the study, completing follow-up, and analysed. Give information separately for for exposed and unexposed groups if applicable.	7
Participants	#13b	Give reasons for non-participation at each stage	NA
Participants	#13c	Consider use of a flow diagram	NA
Descriptive data	#14a	Give characteristics of study participants (eg demographic, clinical, social) and information on exposures and potential confounders. Give information separately for exposed and unexposed groups if applicable.	7, 11

For peer review only

Descriptive data	#14b	Indicate number of participants with missing data for each variable of interest	11, 12, 13, Supplementary appendix tables p1&2
Outcome data	#15	Report numbers of outcome events or summary measures. Give information separately for exposed and unexposed groups if applicable.	7, 11, 12, 13, Supplementary appendix tables p1&2
Main results	#16a	Give unadjusted estimates and, if applicable, confounder-adjusted estimates and their precision (eg, 95% confidence interval). Make clear which confounders were adjusted for and why they were included	7, 11, 12, 13
Main results	#16b	Report category boundaries when continuous variables were categorized	5
Main results	#16c	If relevant, consider translating estimates of relative risk into absolute risk for a meaningful time period	NA
Other analyses	#17	Report other analyses done—e.g., analyses of subgroups and interactions, and sensitivity analyses	7
**Discussion**			
Key results	#18	Summarise key results with reference to study objectives	8, 9
Limitations	#19	Discuss limitations of the study, taking into account sources of potential bias or imprecision. Discuss both direction and magnitude of any potential bias.	9, 10
Interpretation	#20	Give a cautious overall interpretation considering objectives, limitations, multiplicity of analyses, results from similar studies, and other relevant evidence.	8-10
Generalisability	#21	Discuss the generalisability (external validity) of the study results	9-10

For peer review only

**Other Information**			
Funding	#22	Give the source of funding and the role of the funders for the present study and, if applicable, for the original study on which the present article is based	3

Notes:

The STROBE checklist is distributed under the terms of the Creative Commons Attribution License CC-BY. This checklist was completed on 06. October 2020 using https://www.goodreports.org/, a tool made by the EQUATOR Network in collaboration with Penelope.ai

For peer review only
